# Applications of extended reality in pilot flight simulator training: a systematic review with meta-analysis

**DOI:** 10.1186/s42492-025-00206-w

**Published:** 2025-10-23

**Authors:** Alexander Somerville, Keith Joiner, Timothy Lynar, Graham Wild

**Affiliations:** 1 School of Engineering and Technology, UNSW Canberra, Campbell, ACT 2612 Australia; 2 School of Systems and Computing, UNSW Canberra, Campbell, ACT 2612 Australia; 3 School of Science, UNSW Canberra, Campbell, ACT 2612 Australia

**Keywords:** Extended reality, Virtual reality, Augmented reality, Flight, Pilot training, Flight instruction

## Abstract

The use of extended reality (XR) spectrum technologies as substitutes to augment traditional simulators in pilot flight training has received significant interest in recent times. A systematic review was conducted to evaluate the efficacy of XR technologies for this purpose and better understand the motivating factors for this use. The systematic review followed the QUOROM framework (adapted for educational studies), screening 1237 candidate articles to 67 eligible for thematic analysis, with 5 of these also meeting meta-analysis criteria. Existing literature emphasizes the benefits of these technologies, particularly as a result of immersiveness and spatial awareness, enabling the application of more modern educational theories. Although the existing literature is concerned with much of the industry, there is a specific focus on general aviation and the more ab initio skills of flight. The results of the meta-analysis indicate improvements in pilot performance, with an overall meta-analytic effect size estimate of 0.884 (*z* = 2.248, *P* = 0.025), which is positive, statistically significant, and moderately strong. The findings of this review indicate support for the use and intention for the use of XR in pilot flight training simulators. However, multiple serious research gaps exist, such as the potential higher occurrence of simulator sickness and cybersickness, and a lack of robust research trials that examine transfer of training across the full pilot skill set and curricular contexts. This novel systematic review and meta-analysis represent a significant attempt to shape and direct better research to help to direct flourishing technological XR development in a time of increasing pilot shortages and aviation growth.

**Supplementary Information:**

The online version contains supplementary material available at 10.1186/s42492-025-00206-w.

## Introduction

### Context

Computer-based training is an increasingly important part of pilot flight training, and its expansion offers a means of improving pilot flying capabilities and reducing the reliance on aircraft as training devices. Flight training conducted on aircraft is exposed to the same threats and errors as any other flight activity, resulting in significant costs and risks. An analysis of international and Australian flight training indicates that flight training occurrences tend to differ in nature from those of the rest of the industry and occur at a higher rate [1–3]. The costs resulting from Australian flight training safety occurrences, including the inputs required to repair or replace damaged property and the resources expended in life-saving endeavors, totaled AU$250,577,149 (2020 value) in the first two decades of the twenty-first century. Consequently, flight simulations, including various extended reality (xR) technologies, offer the potential to improve efficiency, increase safety, and reduce costs [4].

Recognition of the need for or desire to conduct flight training while not airborne occurred early in the history of aviation. These early flight simulators, such as the Link Trainer, are technologically incomparable with modern high-fidelity simulators. Over many decades of development, simulators have evolved from basic (by modern standards) analog devices to highly complex digital systems that replicate aircraft and their functions in great details. The possible extent and complexity of training have changed, but the fundamental purpose of providing a safer (and cheaper) training environment has changed little. Moreover, even the most advanced high-fidelity simulators, such as those used by airlines, lack the capacity to replicate the immersion and perceptual involvement of flight. This limitation is more pronounced for the lower-fidelity simulators used extensively in the aviation industry. Such simulators are primarily used for instrument flight training and are often merely digital replicas of the flight deck, offering no greater level of immersion than the original Link Trainer. XR technologies such as virtual reality (VR) offer a means of overcoming these limitations by providing far more immersive training environments.

The value of a flight simulator lies in the transfer of skills learned in the simulated environment to the flight environment. This concept, which is known as ‘transfer,’ determines how well the simulator will prepare the trainee for the activity airborne. Such preparation is paramount to reduce the time required for the aircraft to become competent in the task being trained. Although traditional simulators are suitable for training specific procedures, they lack the informational, cognitive, and perceptual fidelity required for training many flying tasks. XR technologies, with their claimed capabilities for immersive, interactive, and information-rich environments, offer a means to augment, or even displace, traditional simulators. Although it is doubtful whether XR, or indeed any other conceivable technology, can fully replace in-aircraft training, it may allow for a significant reduction in the number of hours required in the aircraft.

This study systematically reviews the existing literature on the efficacy of XR flight simulators for pilot flight training, including their potential to augment or replace traditional simulators and the extent to which they can do so. Aligned with established protocols for a systematic literature review, this research examines how the technology can overcome the limitations of traditional simulators, motivating factors for the adoption of the technology, and areas of application within pilot flight training. The two main parts of the research are a thematic analysis to examine current trends in the literature and a meta-analysis to evaluate the efficacy of the technology quantitatively.

### XR spectrum technologies

**Fig. 1:**
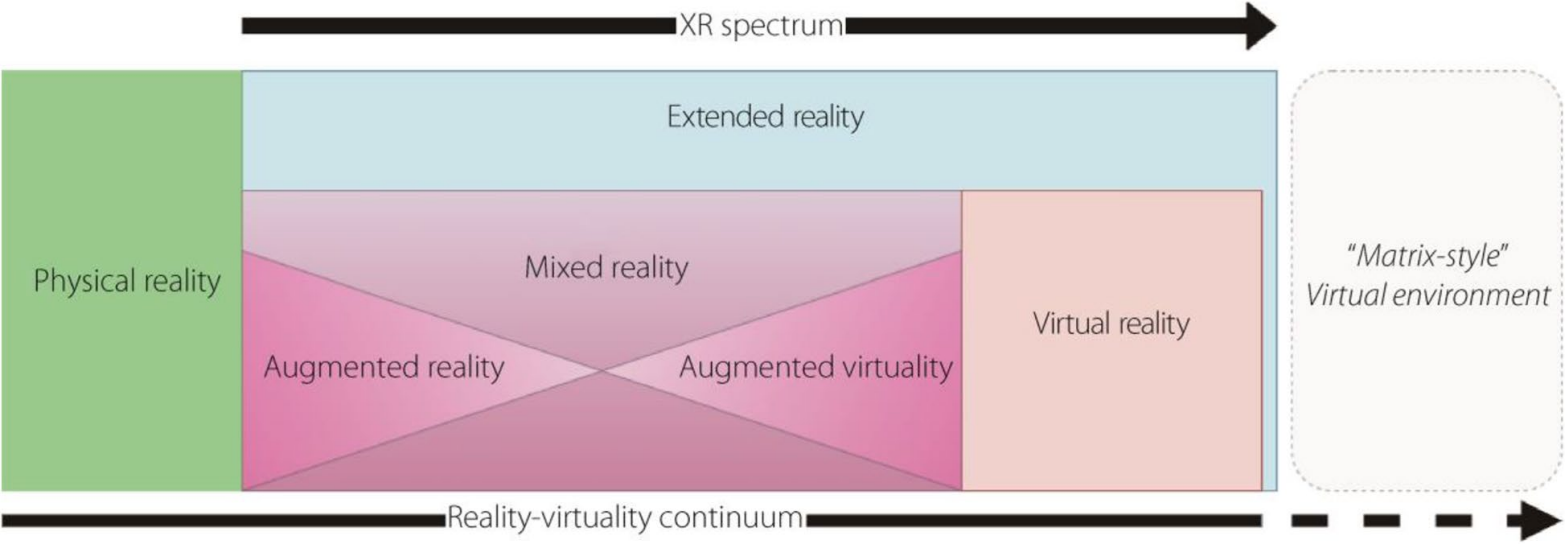
XR spectrum and reality-virtuality continuum diagram and their association [6, 7, 11]

XR describes a set of related immersive technologies, including VR, augmented reality (AR), and mixed reality (MR) [5, 6], as well as emerging technologies between and outside these implementations. The XR spectrum is a more contemporary framework, although strongly related to the reality-virtuality continuum introduced by Milgram and Kishino [7] (Fig. 1). In both frameworks, virtuality is not conceived as a separate category from reality but instead as an extension of reality. Neither the reality-virtuality continuum nor the XR spectrum interprets virtuality as being opposite to reality; in contrast, there is a gradient from reality to virtuality. These technologies immerse users in computer-generated environments across a spectrum of sensory isolation. The purpose of this immersion and the extent to which it is achievable vary across the XR spectrum. At the lowest level of immersion, the purpose may be the overlay of entertaining visual elements [8] or data that are critical for the performance of surgery [9, 10]; at the highest level of immersion, the goal can be full isolation within a computer-generated environment with a negligible relationship with the physics or visuals of reality [11].

VR, which is the closest to full virtuality of primary XR implementations, refers to a set of technologies that enable the experience of a ‘fully’ immersive, three-dimensional (3D), computer-generated world. The concept of VR has existed for decades, with a flight simulator sometimes regarded as a form of VR, particularly prior to the emergence of, and wider association with, immersive computer-generated environments. The work of Mandal [12] more closely resembles and is considered the origin of what is currently considered as VR. For a technology to be considered as VR it must include 3D computer-generated graphics that create a simulated environment [13], incorporate real-time feedback and response to actions undertaken by the user, such that they ‘interact’ with the environment, and provide a sense of immersion within the environment to the operator. This is generally achieved using a head-mounted display (HMD), which provides a fully synthetic environment with no intentional merging of real and virtual environments [14]. Systems that offer any intentional merging of real and virtual worlds, whether through an HMD or not, are classified as MR or AR [7].

AR, which is further from full virtuality than VR, integrates virtual elements into the real world. Such systems merge real and virtual elements in a real environment while maintaining alignment between each object, enabling 3D freedom of movement in real space and real-time interactivity [15]. That is, the environment is not computer generated; rather, the real world is enhanced with computer-generated elements [16]. The elements imposed on the real world may be pure information, usually in the form of text, or intricate photorealistic items that are designed to be indistinguishable from real objects [17]. As noted by Lallai et al. [18], the inherent interactivity of AR offers unique benefits for supporting training that are not available in more traditional systems. AR technology may take the form of an HMD, but more often than VR, it may use existing general-purpose, personal, and portable computer devices. Although the use of mobile phones to access VR has achieved some success, such as Google Cardboard [19], these implementations tend to achieve less immersion than discrete hardware. However, it is far more common to access AR on general-purpose hardware such as viewing products for sale online [20].

MR varies depending on the specific implementation and may be located at almost any position on the reality-virtuality continuum. MR combines AR and VR to create hybrid virtual-physical environments [21]. This combination can be achieved either by superimposing computer-generated virtual objects that interact with the real world, or by enabling real-world objects to interact with the virtual environment. MR represents a large part of the continuum between the purely real and wholly virtual, including AR as a constituent subset (Fig. 1). While MR is the latest XR technology to receive significant research interest and undergo growing public use, such technology has arguably been used within flight simulation for decades. Full flight simulators (FFSs) use an analog of an aircraft cockpit, with flight experience represented by the display of moving imagery within the scene, either on flat-screen monitors or projectors. Therefore, such an FFS is logically a primitive form of MR, although MR has not been used in the literature when referring to FFSs. In contrast, VR has undergone a definite change with the continuing development of technologies. The mixing of real and virtual elements allows for direct interaction between the trainee, real elements, and computer-based system [22]. The virtual media element represented by the MR hardware can support the cognitive learning process [23]. The technology that enables MR is often the same as that for AR and VR.

Some studies also consider cave automatic virtual environment systems as a form of VR [24], where real-world controls usually exist within the virtual space. However, because such a definition would necessarily expand the scope of the present research to devices such as FFSs, these systems are not considered. HMDs used in VR are separate hardware devices worn by the user and consist of a headset. Within the headset, a combination of screen(s), and often lens(es), is placed in proximity to the eyes. The illusion of depth, which is necessary to achieve immersion, is created through parallax. The human perception of parallax is similarly exploited in spatially or temporally multiplexed display systems. Whereas multiplexed systems alternate left and right views or superimpose these views onto the same display, VR HMDs show both views simultaneously split between each eye [25]. Within the headset, each eye can see only one view; the left and right view being isolated.

HMDs designed for AR are of the ‘see-through’ type, as opposed to the ‘immersive’ type required for VR. See-through HMDs may be further distinguished by the means by which the user can see through to the real world: optical or video see-through [26]. Optical see-through HMDs (OST-HMDs) use semi-transparent mirror(s) positioned in front of the eyes, through which the user views the real world. The arrangement and design of these semi-transparent mirrors vary significantly among devices, with the half-mirror, birdbath, and free-form prism being notable examples [27]. The mirrors reflect computer-generated content, resulting in the merging of real and virtual elements. Because the optical interface of such a device is simply a thin parallel plate, only a small number of optical aberrations are introduced into the scene. This setup means that the resolution of an OST-HMD system is almost as good as that of the human eye [26]. Video see-through HMDs (VST-HMDs) use external cameras on the headset to capture a video of the real world and then stream it to the user through the internal optics and display system. Computer-generated content is incorporated into the video stream in real time, resulting in the apparent integration of virtual elements into the real world [28]. This arrangement means that the physical hardware of a VST-HMD may be the same as that of an immersive HMD, such as in the case of ‘Passthrough’ on more modern Meta XR devices [29]. A potential advantage of VST-HMDs over OST-HMDs is that the real-time manipulation of the video stream allows for greater flexibility in the environments that can be used. However, the resolution of the real world is inherently limited by the resolution of the camera(s), HMD display, and HMD optics.

**Fig. 2:**
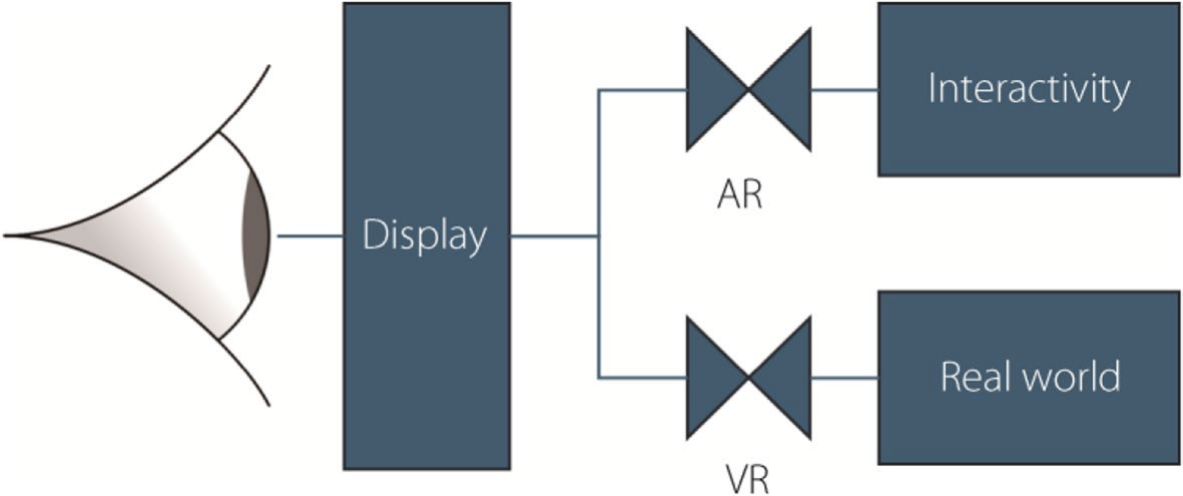
Representation of real-world and interactivity modulation i3n XR devices

The most readily available and prevalent types of XR technologies are general-purpose handheld devices, such as smartphones or computer tablets [30]. Underlying handheld XR is VST, which is based on the same working principles as VST-HMDs: the camera of the device captures a video of the real world, virtual elements are added to the stream, and the merged virtual and real elements are presented to the user on the screen. This is illustrated in Fig. 2 as the two flow control values; one for elements from the real world (no real-world input being VR) and the second controlling the level of interactivity (zero interactivity being AR). Interaction in XR is achieved by both the ability to move the device in 3D space and by the touchscreen. The ubiquity of such interactions is enabled by the basic components of VST being present on almost all modern mobile devices: cameras, sensors, processors, and screens [31].

The primary input hardware required for any XR device is a sensor(s) that enables accurate tracking of the free movement of the user/device in 3D real space in real time. For accurate position detection, devices generally use optical, magnetic or inertial tracking, or a combination thereof [32]. Optical tracking uses the camera(s) on the exterior of the device coupled with image processing to detect and track features of the surrounding environment or specific tracker markers within the environment, allowing for the location and orientation of the device to be determined relative to the surroundings. Magnetic tracking generally relies on an internal magnetometer to orient the device by detecting changes in the Earth’s magnetosphere [33]. Inertial tracking uses accelerometers and gyroscopes to detect acceleration in the three axes of motion and rotational velocity around the same axes to calculate the position and orientation [32]. Other XR devices may use more niche tracking solutions, either separately or in addition to more commonly used tracking technologies. For example, some modern devices, such as those manufactured by Apple Inc., use Light Detection and Ranging technology to increase the tracking accuracy [34].

The secondary input hardware in XR devices typically consists of sensors and devices that facilitate interactivity [28]. The most basic type of secondary input technology is a mouse and keyboard, which may be suitable when full immersion is not attempted. However, technologies such as hand controllers, gesture recognition, motion-capture wearables, and touch screens offer far greater interaction. Hand controllers are handheld devices that track the position of the user’s hand and usually consist of sensors, buttons, triggers, joysticks, and sometimes haptic feedback [28]. By tracking the controllers, the positions of and intended interactions with the user’s hands can be translated into the virtual environment. Gesture recognition attempts to track and interpret user movements, particularly those of the hands, by using cameras, depth sensors, and motion sensors. When implemented to track the hands, gesture recognition serves the same purpose as hand controllers but without the additional hardware and intermediary translation of intention. Motion capture wearables vary in form factor and intended use and may be represented by a single wearable sensor, a single garment (e.g., a glove), or a full suit, which can translate the user’s body and motion into the XR environment [35].

In terms of the application of XR to pilot flight simulator training, an additional input hardware category must be considered: specialized flight peripherals. Flight peripherals are highly dependent on the class and category of aircraft to be simulated, the training outcomes and qualifications sought, and the resources available. The most distinct peripherals required are flight controls. A combination of a joystick, yoke, throttle quadrant, collective, cyclic, and pedals is required for an accurate representation of the simulated aircraft and for realistic control of the aircraft [36]. Various secondary aspects of the simulated aircraft may be preproduced, such as the flight instruments, control panels, circuit breaker panels, communications and data equipment, and even a realistic representation of the cockpit surrounds. In traditional simulators, these secondary aspects of the simulated aircraft are commonly reproduced virtually and viewed through a computer monitor. With XR, it may also be possible for both the visual of and interaction with the flight controls to be achieved entirely virtually.

### Pilot education and flight simulators

The process of becoming a pilot, particularly a commercial or air transport pilot, involves acquiring the requisite knowledge and practicing technical and non-technical skills (NTSs). The aspiring pilot must first select the aircraft category, where ref. [37] defined the six aircraft categories as airplane, helicopter, power-lift, gyroplane, and airship. The license holder must generally hold a suitable class rating from the five available options: single-engine airplane, multi-engine airplane, single-engine helicopter, single-engine gyroplane, and airship [37]. In civilian aviation, there are four additional levels of license that a person can hold: the recreational pilot license, private pilot license, commercial pilot license (CPL), and air transport pilot license [38]. The required hours, skills, and standards increase significantly as higher licenses are acquired.

Within the Australian context, the required knowledge is categorized into seven areas: human factors, aerodynamics, flight rules and law, aircraft general knowledge, meteorology, navigation, and flight planning [39, 40]. The knowledge requirements for Australian pilots at each training level are defined in Schedule 3 [41]. Each level of license, as well as the various ratings, requires a minimum number and types of flight hours. During the ‘building’ of these hours, it is necessary to acquire certain skills. These skills, which are specified in Schedule 2, include those required for all pilots and those only applicable to certain specializations [41]. The acquisition of technical and NTSs, the learning of substantial knowledge, the qualification for a certain rating, and even the building of certain types of flight hours can be augmented in a suitable and often accredited flight simulator [37].

Flight simulators generally offer four distinct advantages over training in real aircraft: increased scenario controllability, reduced risk, greater repeatability, and reduced costs [42]. The training of certain maneuvers and emergencies in a real aircraft represents an unacceptable risk to both pilots and aircraft. However, in the controlled and safe environment of a flight simulator, these scenarios can be practiced. The experience within a flight simulator can be well controlled by the simulator instructor, depending on the fidelity of the simulator. As such, the conditions and sequences can be made identical over multiple flights or simplified to assist in training, which is typically difficult and sometimes impossible in the real world. The cost differential between the flight simulator and aircraft, whether in the initial acquisition, maintenance, or operation, usually significantly favors the simulator, which in turn allows for much greater access. For a flight simulator to be of the greatest usefulness, it must adequately mimic the physical and environmental conditions. In some cases, replication is achieved through a combination of hardware and software, including a motion system that attempts to reproduce aspects of the accelerations felt during crewed flight and aircraft maneuvers [42].

The hardware most often includes a combination of the specialized flight peripherals already highlighted and a computer to link these components to suitable software [43]. With the rapid increase in computer power and the continued decrease in the cost of processing power, many modern consumer computers and even consumer-level portables are capable of operating as flight simulators. However, high-end graphics cards and CPUs are required, particularly when the highest visual fidelity of the simulated environment is required or when the model and motion fidelity are important. Some flight simulators, and most FFSs, use a motion system, including acceleration and deceleration, as well as pitch, roll, and yaw. The fidelity of the motion produced and the need for motion are matters of continuous research [44, 45]. The visual system of a flight simulator provides the necessary imagery to the crew, usually using flat screens, curved screens, flat or dome-like panels onto which a projection is shone, or a combination of these technologies. The quality, design, and capacity of each hardware component will impact the fidelity and ‘immersiveness’ of the simulator for the participant.

A flight dynamics model is a mathematical representation of the behavior and flight characteristics of the aircraft [36], and modern simulators make use of such numerical and stability derivative methods [46]. The underpinning database system contains information such as the geometry and specifics of the simulated aircraft, terrain, and airport data, as well as object and instrument data. The database system retrieves the necessary data, which the image generation system then combines with additional information and presents to the crew via the visual system. For a simulator to be useful for flight training, it may feature processing software that includes a weather generation system, air traffic control (ATC) simulation, and an instructor operating system [47]. Weather generation systems based on probabilistic models create sufficiently representative wind, turbulence, precipitation, and visibility variations in the simulation [48]. The ATC simulation software component simulates the interactions and communications between pilots and ATC, sometimes including background radio chatter, to provide a realistic radio environment [49].

Within general aviation (GA), flight simulators have traditionally been used for basic instrument flying training during the early stages of training as well as instrument rating training. Time in the simulator reduces costs by (1) limiting the time required in the aircraft, (2) enhancing the value of time in the aircraft by allowing additional learning prior to application, and (3) providing practice flight hours. Within regular public transport, flight simulators serve a very similar purpose to that in GA, but with the additional opportunity to complete certain qualifications fully without time in real aircraft. Recreational aviation, as both the newest and lowest-cost aviation sector, has generally not made any prescribed or logged use of flight simulators. However, within all sectors of aviation, the ubiquitous availability and decreasing cost of computers has meant that trainees arrive for training “…fully training up on [Microsoft] flight simulator…” [50]. That is, the use of basic flight simulators with consumer-grade computers is now an inherent part of the pilot training experience. Many civil aviation regulators, including the FAA [51], now accept the use of personal computer aviation training devices (PCATDs) for the training of certain skills under specific conditions. Current technology trends in flight simulators, including their use in place of traditional flight hours at all levels, are largely owing to the cost differential and available fidelity of such devices. Therefore, technologies that can reduce costs further and increase fidelity are likely to prosper.

MR, VR, and AR have all been utilized as training tools in research as well as in various commercial training situations [52]. The design and implementation of flight simulator pilot training has received significantly less research attention than other applications [53]. Questions remain regarding whether the overlay of information, prompting of exterior scan, overlay of flow patterns, and inflight use of the technology are safe and effective [54]. Research in other domains has shown that XR technologies offer time and cost savings in various training situations [55], although their efficacy is yet to be established for more complex, real-time tasks. VR technologies are being researched for miniaturization and dematerialization of traditional simulators, resulting in more immersive and less expensive solutions [56]. AR and MR have yet to receive the same volume or depth of research as VR in the flight training domain, although some research has been conducted on the implementation of AR in the education of aviation maintenance personnel [57].

In the cockpit training environment, whether simulated or present, there are multiple opportunities for the addition of virtual elements to prompt and coach correct piloting techniques. Existing literature on aviation maintenance training using AR shows a positive ability to draw trainees’ attention to critical elements at specific times [22].

## Methodology

The research question for this review was: “What is the effectiveness of XR technologies as an adjunct to, or full replacement of, traditional simulators in pilot flight training, and what are the motivating factors for its use?” Therefore, this review is limited to the pilot training applications of XR flight simulators and not the development of such simulators. This review is limited owing to the presence of existing systematic reviews that consider the implementation, but not necessarily the application, of XR flight simulators [58]. The present review was designed, conducted, and reported in general conformance with the Quality of Reporting of Meta-analyses (QUOROM) statement [59], with the quality of the papers included in the meta-analysis further assessed using the consolidated standards of reporting trials (CONSORT) checklist as is suitable for educational studies [60]. The QUOROM statement checklist is provided in Appendix D as additional material. The CONSORT checklist is provided in Appendix C as additional material.

**Fig. 3:**
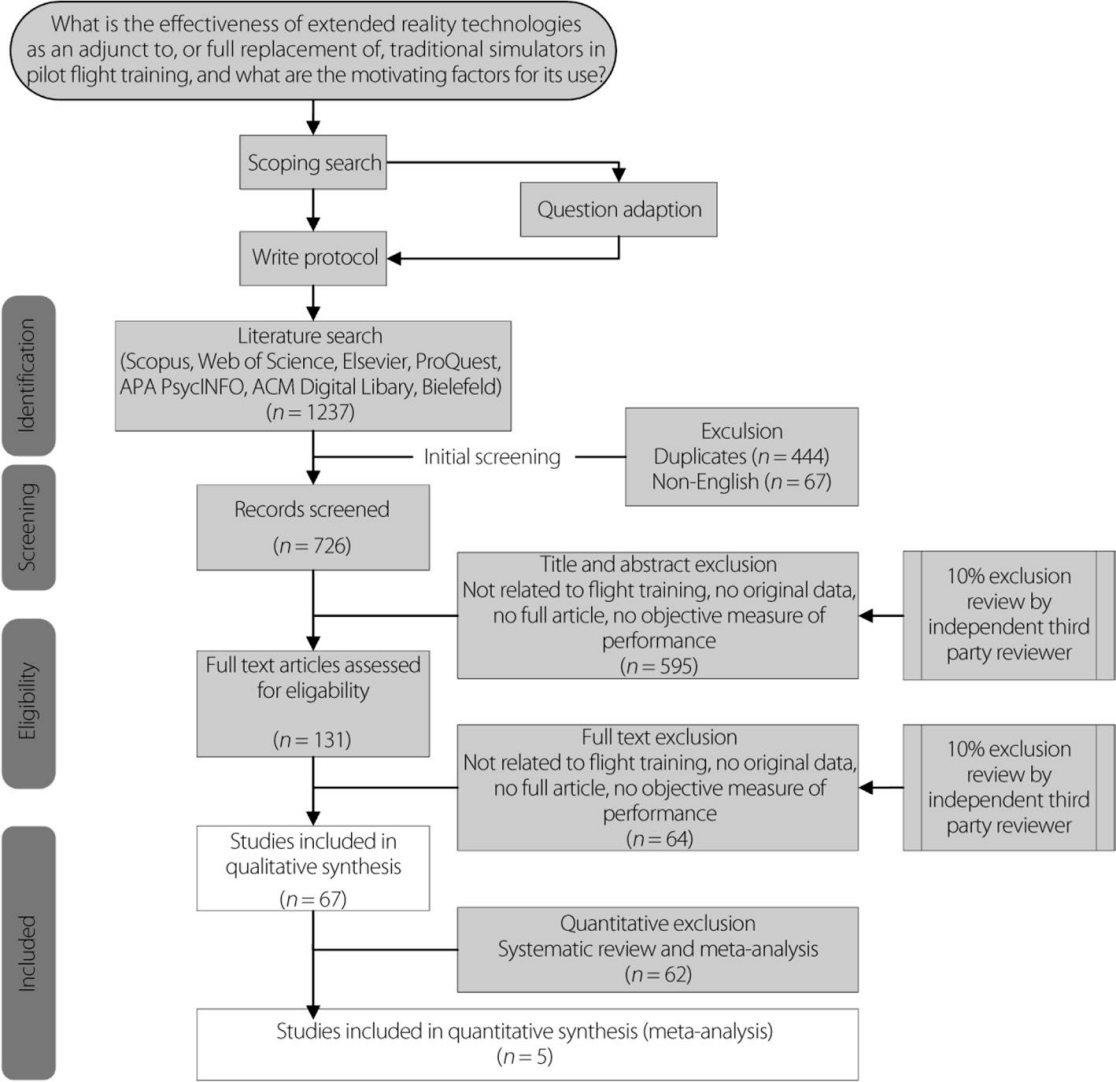
Systematic process of review of the literature

The review included randomized controlled trials (RCTs), non-randomized comparative studies, and concept papers reporting the use of VR as an adjunct to pilot training or as a replacement of traditional simulators for the transfer of flight critical skills. Seven databases were searched to identify these studies: Scopus, Web of Science, IEEE Xplore, ProQuest, APA PsycINFO, ACM Digital Library, and the Bielefeld Academic Search Engine. The search was conducted in September 2022 and included all studies published since the inception of the databases that met the search criteria. The systematic search of these databases used terms consisting of a primary phrase specifying the section of the XR spectrum of concern and a secondary group of phrases pertaining to pilot flight training, as listed in Table 1. An initial screening was performed by one researcher to remove duplicates (*n* = 444) and studies that were not published in English (*n* = 67). This process is illustrated in Fig. 3.

**Table 1:** Systemisation and combination of search terms

Primary term	Secondary term
Virtual reality	Flight training
Virtual reality Virtual environments HMD simulation	Flight instruction Pilot training Pilot instruction
Mixed reality Augmented reality Extended reality	Aircraft simulator Flight simulator

For this analysis, the inclusion criteria were: (1) directly related to flight training, (2) contained original data or original concepts, (3) implemented an XR solution in a training context, (4) concerned with pilot training, and (5) reported on a performance or usability outcome. Here, flight training is restricted to training for operating aircraft, whether crewed or remotely piloted. Based on the researchers’ experiences and judgment, any research that lacks a direct evaluation or focus on pilot training should focus on an aspect of XR flight simulation with an inherent and critical application to such training. When the first reviewer identified publications for inclusion, a discussion was conducted with the second reviewer to confirm that the link was adequate. For inclusion in the thematic analysis, a specific motivation for the use of the technology had to be evident in the research. For inclusion in the meta-analysis, sufficient data had to be reported in the research to calculate the Hedges’ *g* effect size(s) as the group mean and standard deviation or the standard error data and number of subjects in each group, for both the reported control and intervention groups.

The first-stage screening of the search results by title and abstract was completed by one reviewer only. A second reviewer then screened a random sample of 10% according to the method suggested by Torgerson [60]. Studies that did not meet the inclusion criteria or were not available as full texts to the reviewers were excluded. The agreement between the first and second reviewers in terms of a random sample of citations was then used to calculate the inter-rater reliability. The Cohen’s kappa statistic was subsequently calculated to quantify how well the reviewers agreed [60, 61] compared with chance. The agreement was high (κ = 0.877) and the results of the first reviewer were found to be reliable.

Second-stage screening of the search results for identifying research suitable for inclusion in each analysis and the exclusion of irrelevant research was completed by reviewing the full text, again by one reviewer only. A second reviewer then screened a random sample of 10%, and the Cohen’s kappa was subsequently calculated to quantify the agreement between the reviewers. The agreement was again high (κ = 0.857) and the first reviewer was deemed reliable.

Narrative content coding for all studies for inclusion in the thematic analysis was completed by one researcher, establishing codes a posteriori, with the second researcher verifying grouped primary subjects. Data for all studies for inclusion in the meta-analysis were extracted by one researcher and were checked by the second reviewer using standardized data tables established a priori. Critical evaluation of the level of evidence of the included studies was assigned with reference to the guidelines developed by refs. [62, 63]. Where such an assessment was appropriate, the study quality was evaluated using the method specified in Section Six of the Cochrane Review Handbook [64]. Such an evaluation was based on multiple parameters: the quality of the reporting of the study methodology, blinding of the outcome assessors, sample size, and, in the case of RCTs, the process of randomization and allocation concealment.

Thematic analysis was conducted using LibreOffice Calc (Version 7.3.5) [65]. Codes were developed to represent common features and underlying ideas such as technical limitations, reported issues, or stated motivations. As the trends and commonalities of the codes became apparent, the codes were grouped into primary subjects. An explicit analysis of these codes and subjects was possible by comparing the frequency, identifying the co-occurrence, and relational sets. Therefore, although the data were qualitative insofar as they did not indicate ordinal values [66], the type of analysis was quantitative [67]. This goes further than a simple thematic analysis by organization and description [68], and allows the interpretation of various aspects of the topic [69]. Meta-analysis was conducted using *JASP* (Version 0.16) [70], followed by data formatting in LibreOffice Calc (Version 7.3.5) [65]. The Hedges’*g* effect sizes [71] were calculated for each study, with the mean differences between trial performance outcomes for the intervention and control groups divided by the pooled standard deviation, as opposed to the maximum likelihood estimator [72]. Hedges’*g* is often preferred in meta-analyses, especially when studies with varying sample sizes are combined, owing to its ability to adjust for small sample bias [73]. For studies that reported multiple end-points or outcomes, a single effect size was calculated using the method recommended by Borenstein et al. [74]. In this analysis, the correlation coefficient between the group means for the different outcomes across the samples within each study was used to reflect the correlation between the effect sizes, under the assumption that the difference between group means was representative of the underlying relationship between multiple outcomes.

The null hypothesis (*H*_0_) for this meta-analysis was that there would be no effect resulting from the educational intervention, such that the random-effects model would show a Hedges’ *g* of zero. Conversely, the alternate hypothesis (*H*_1_) was that there would be a significant overall effect, resulting in a non-zero value of Hedges’*g*. Therefore, the null hypothesis is as follows:

$${H}_{0}: {\mu }_{i}= 0\ for\ i=\left\{objective, subjective\right\}$$

where *μ* is the difference in outcomes—either objective or subjective—between control and intervention, with zero indicating no effect resulting from the intervention. The alternate hypothesis is as follows:

$${H}_{1}: {\mu }_{i}\ne 0\ for\ i=\left\{objective, subjective\right\}$$

where the difference in outcomes, either objective or subjective, between the control and intervention is not equal to zero. A non-zero outcome indicates a significant effect of the intervention, whether negative or positive, with a negative value representing deterioration.

These data were analyzed separately to ensure that the subjective and objective measures did not interfere with the detection of trends in the other. The objective data were direct measures of flight performance and tasks that are highly critical to and linked with the task of piloting an aircraft. The subjective measures were those based on individual perception, or other measures wholly removed from flight (hereafter referred to as subjective), and are quite different from the technical and operational skills of piloting an aircraft. Based on the reviewers’ general familiarity with similar research literature, the assumption was made that different studies would estimate different, yet highly related, intervention effects. Therefore, a variant of the inverse variance method was used to produce a random-effects meta-analysis. The well-established DerSimonian-Laird method [75] was used, wherein the study-specific estimates of the standard error (Se_i_) are adjusted to integrate an amount of the degree of variation (τ^2^), as observed among the intervention effects in separate studies [76]. The intervention effects and standard errors of the studies included in the analysis were used to assess the tau-squared values (τ^2^). The guidelines set forth by Cohen [77] were adopted for interpreting the magnitude of the effect size. That is, an effect size greater than 0.65 was considered large, that within the range of 0.35–0.65 was considered moderate, and that within the range of 0.2–0.35 was considered small. 95%CIs were used for statistical inference.

## Results

A total of 1237 studies applying XR technologies to pilot flight simulator training were identified through the literature search of the seven databases. Among the articles identified, 444 were found to be duplicates and 67 were not written in English. After the initial exclusion, 726 articles remained.

During the first stage of screening, based on a review of the titles and abstracts, the first reviewer reliably excluded 595 articles and included 131 articles for full-text review. The use of ‘pilot’ in the secondary term led to the capture of pilot studies, and therefore, resulted in a high rate of exclusion. During the second stage of screening, based on a review of the full text, the first reviewer reliably excluded 64 articles and included 67 for data synthesis. The full text of two articles [78, 79] was not available to the reviewers at the time of review. Of the articles included in the data synthesis, all 67 were identified as suitable for inclusion in the thematic analysis and five were identified as suitable for inclusion in the meta-analysis. None of the included articles was an RCT. The articles included were primarily concept papers (46%), surveys (10%), and non-randomized or quasi-randomized comparative trials (43%). Appendix A presents a general description of each included article, along with the corresponding findings.

**Fig. 4:**
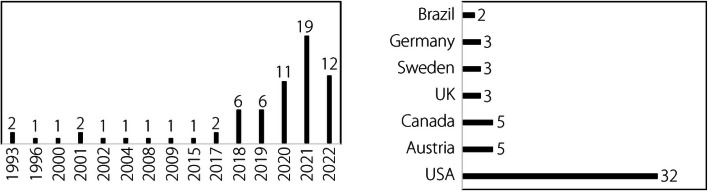
Year of publication of included articles (left); origin of included articles (right)

The year of publication of the included articles is an important factor, particularly given the impact of evolving information technology on both flight simulation and XR as well as the increasing knowledge on how learning and training are achieved. Given the increasing power and portability of computers [80] and the widely publicized release of consumer-grade HMDs [81, 82], among other factors, the increasing number of publications in this area of research is understandable. Figure 4 shows the number of years since publication of the articles included in this review. More included articles were published in the past three years (63%) than in the past 16 years.

There are clearly two phases in XR research: the early exploratory period from 1993 to 2015 and the growth period from 2017 to 2022. The early period was characterized by only one or two papers per year, with only nine of the 24 years from 1993 to 2016 having publications. The growth period showed statistically significant linear growth (r = 0.954, *P* = 0.003, *n* = 6) and exhibited a growth rate of 3.5 articles per year. Note that 2022 was normalized with data for eight of the 12 months, resulting in an estimated 18 articles per year in 2022.

The origin of the research can also be important, as cultural, economic, and socio-political differences can impact the outcomes and accurate reporting of outcomes. The present review only included articles published in English, and as such, may be susceptible to certain biases. Figure 4 shows the origin of the articles included in this review from the top seven countries. More articles originated from the United States than from almost all other nations combined (49%). Research has shown that varying pilot training patterns and demands among nations may drive such research patterns [83].

### Thematic analysis

**Fig. 5:**
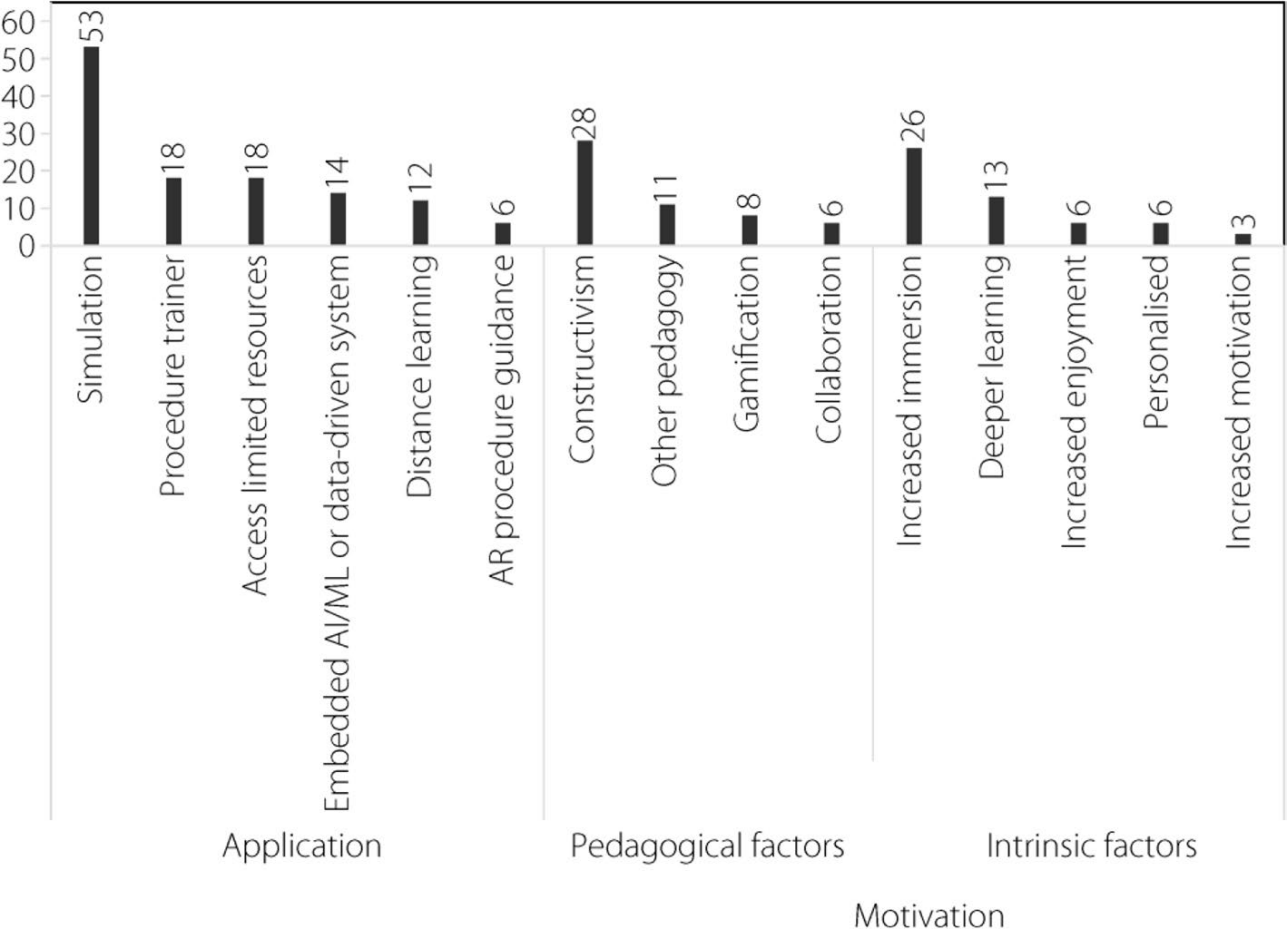
Applications and motivations

A thematic analysis was conducted on all 67 final articles, focusing on six identified applications (Fig. 5), each contained within the domain of the research question. Individual articles may or may not possess the potential to be defined by multiple classifications within the grouping and sub-groupings of codes, resulting in cumulative totals of codes greater than the total number of articles (see Appendix B – Tabulated Thematic Analysis Data). Thus, as coded, the application count of 124 articles exceeded the 67 articles included in this review.

#### Application

Simulation was the most commonly coded application type. The use of XR for procedure trainers and to enable access to limited resources were frequently identified applications. Procedure trainers are devices or software that allow the practice or training of specific flight procedures and protocols. Procedure trainers have traditionally been employed for training and practice of the procedures needed for instrument flying [84]. The use of XR for application as procedure trainers is often an enabler of access to limited resources, as it can replace larger existing hardware solutions. Füchter et al. [85] further emphasized that such systems, including the system presented in their article, “…can be used anywhere without the need for computers, screens, joysticks, or any other hardware.” The move from traditional simulators to lighter and more portable XR solutions, for both procedure trainers and FFSs, can be classified as a type of dematerialization. Advances in technology have resulted in a “decline over time in weight of the materials used in industrial end products” [86]. XR training flight simulators, and more particularly VR simulators, primarily make use of commercial off-the-shelf (COTS) headsets [24, 87–90], hardware [85, 91], and software [14, 92], which are physical and financial enablers of access to limited resources.

The facilitating reduction in the mass of an XR simulator and the increasing power and efficiency of microprocessors enable its application in distance learning. Distance learning, which was the application of 12 articles, refers to education undertaken by students at a location remote from the educator and, in recent times, largely facilitated by the Internet [93]. The mechanism that enables distance learning is often an extension of the existing remote education paradigm and uses the same underlying infrastructure, such as cloud-based video conferencing [94]. Distance learning may offer greater flexibility to students [95], but also enables more efficient use of a particularly limited resource – qualified flight instructors. Therefore, distance learning architectures for flight instruction should assist the instructor, and not simply improve the convenience of the learner. For example, the instructor can view the actions of the learner within the virtual space or automate basic instructional tasks [96]. Given the continuing pilot shortage [97] that necessarily affects the flight instructor workforce, applications that reduce the time investments required by instructors are important.

Applications such as AR procedure guidance and embedded machine learning (ML) or data-driven systems were present in 14 and six articles, respectively. AR procedure guidance is the use of AR to guide trainee pilots through specified flight procedures, achieved using both general-purpose handheld devices [98] and see-through HMDs [18, 56, 94]. Notably, all studies that used discrete AR hardware employed OST-HMDs; none made use of VST-HMDs. The AR procedure trainer can also serve as a high-fidelity replacement for procedural documents for the training of procedures in spatially complex environments such as cockpit flows [56].

For such an AR procedure trainer, or indeed, a different XR flight training simulator implementation, to be of the greatest functionality, the automatic retrieval and processing of data are highly advantageous – that is, an embedded ML or data-driven system. The provision of automated feedback was most often the goal of the examined research: either feedback about performance and learning provided to the learner [96, 99] or feedback about the same provided to help the instructor to alter the teaching provided [92]. In such implementations, in which feedback is provided directly to the learner, the human flight instructor can be said to have been replaced by a “virtual aeronautical instructor” [96]. As opposed to basing feedback purely on flight performance metrics, as is common in both XR and traditional training simulators, ‘bio’ data can be used. Such data are mostly derived from various types of sensors on or near the learner, such as those in ref. [100]. Camera-based eye tracking and galvanic skin response are two common potential approaches [101]. Although the literature examined most often focused on the provision of feedback to and for the learner, the data themselves can have post-training educational purposes. That is, the data retrieved and processed by one of the various modern techniques can inform the design of the system and the instructional approach. The second category of embedded ML or data-driven systems within the included articles is based almost exclusively on biosensor data. For example, the data from eye trackers can be used to assess the expertise of a pilot [70] with a greater level of objectivity that is otherwise possible. Similarly, the use of various types of sensors and techniques to collect signals from the central nervous system can better track training progress [100]. The increasing use of data in instruction, particularly in simulation-based instructional systems, is not limited to XR (e.g., [102]); however, the proximity of the hardware used to the learner may provide an advantage. To this end, many COTS embed the hardware and software required for eye tracking [90]. Other biosensors are less commonly embedded in such systems, and certain sensors have known limitations in VR applications [103].

#### Pedagogical factors

The motivations for using XR simulators in pilot flight training can be divided into pedagogical and intrinsic factors (Fig. 5). Pedagogical factors are theories and considerations that impact the learning process, including the instructional techniques, assessment methods, and learning environment. Constructivism, which was present in 28 articles, was the most common pedagogical approach cited as motivating the adoption of XR technology. Constructivism, which is one of the five main pedagogical approaches, is an educational theory that stresses that learning “is constructed from, rather than being a direct response to, the stimulus need for active engagement” [104]. That is, learners must be actively engaged in constructing their own knowledge and understanding through experience, rather than knowledge being absolute and solely a matter of transfer. The prevalence of constructivism in XR literature contrasts somewhat with that of traditional flight training, including traditional flight training conducted in flight simulators. Instructivism, which focuses on the direct transmission of knowledge from the instructor to the mind of the learner [105], has been and continues to be the approach used almost exclusively in the training of pilots. The instructivist approach requires the formation of associations with stimuli, largely through drills and memorization, with less consideration for the unique construction of knowledge by the learner [106]. In this manner, instructivism aligns with the principles of deliberate practice [107]. In the context of flight training, this approach has clear advantages, particularly for acquiring accurate psychomotor skills and procedures. Learning theory has changed over time, moving progressively from instructivist to constructivist [104] and is currently shifting towards connectivism [108]. Flight training has largely maintained the older learning approaches. However, modern flight instructor literature acknowledges a fourth level of learning, namely correlation, which recognizes that the learner will construct an understanding through experience [109]. The emergence of the PCATD based on inexpensive yet powerful personal computer devices and the newer XR technologies allows for the creation of ‘microworlds’ in which learning can take place, while requiring far fewer resources. These microworlds “offer an interesting compromise between the instructivist and constructivist approaches” [110], and allow greater opportunities for learners to be active in creating understanding through hands-on experience.

In the examined articles, the constructivist approaches included interactivity, scenario-based training, self-directed learning, and real-time feedback. The use of interactivity and scenario-based training exists in traditional simulators but is further expanded by the capabilities of XR technology. Interactivity during learning, which facilitates constructivist principles of active engagement and safe experimentation [111], is an inherent part of airborne flight training. The constructivist interactivity of an XR flight training simulator is largely owing to its ability to enhance digital and non-digital ground training tools. For non-digital ground training tools, such as the “paper tiger” and ground-based procedure trainer, AR can overlay interactive elements [98]. Such augmentation of non-digital tools can allow for real-time feedback, which is not otherwise available. At a more fundamental level [13], XR must facilitate real-time feedback in response to the actions undertaken by the operator. The operator in the XR system, who is the learner in this case, has additional opportunities to direct their own learning. In this manner, the learner can construct their own knowledge and understanding through experience.

Digital flight simulators most often make use of flat-panel screens, which limit learners’ understanding of spatial relationships, particularly when such understanding is derived from visual information (i.e., visuospatial). More complex FFSs make use of visual systems that enable a greater visual range [112]; however, these are expensive and do not necessarily achieve the immersion made possible by HMDs and XR [113]. The real-time tracking of head movements of HMDs [18], which increases immersive interactivity, can enable more effective emergency training [114]. The use of traditional simulators for scenario-based training of emergencies that are too dangerous to undertake in real life is common, and this is made more interactive using XR hardware. Emergency training using XR is also well established in other safety-critical industries [115, 116]. Scenario-based training in XR need not be restricted to emergencies, but can include activities that are impractical to teach on the job [117]. Under the traditional flight training paradigm, the training of emergency procedures requires the presence of a suitably qualified instructor, which can be changed using XR. For example, using XR and incorporating game mechanics, Lekea et al. [118] constructed an “escape room” for emergency training without the need for an instructor.

Gamification and collaboration motivated the pedagogical factors in eight and six articles, respectively. A collaborative approach involves groups working together to construct shared knowledge. Learners within the groups actively participate in solving problems through collaboration, and in so doing, promote social interaction, the non-threatening sharing of new concepts between learners, and refined knowledge construction for the individual learner and other members of the group [119]. Such learning has been shown to be socially reinforcing in other contexts and can lead to better knowledge retention [120]. Most of the existing training that pilots undergo is best characterized as instructivist, although the use of a collaborative approach is not without precedent. Within the syllabus for the training of flight instructors, at least in Australia, is the opportunity for collaboration during “mutual flying” practice [121]. This mutual flying practice involves two flight instructor candidates who already hold suitable licenses practicing training exercises and collaborating to construct a shared knowledge of the art. Collaboration in the context of XR may use traditional techniques such as intragroup collaboration [122] and role-play [118], or new methods enabled by XR technology. However, while such XR-enabled methods were hinted at in the included studies, none implemented non-traditional collaboration.

Gamification, which may utilize collaboration, involves integrating game elements into the learning process. Point scoring, rewards, and challenges are included in the learning process to enhance learner engagement and knowledge acquisition. Although such elements may naturally exist within training programs, as in many human endeavors, they are not necessarily intentional. In contrast, such elements are arguably taught against in many human factor courses [123]. Gamification may represent a pedagogically valid approach for enhancing safety-related training within flight simulators or XR-based flight simulation environments. Implementations of gamification within the included articles include “escape room” games [118] as well as the incorporation of guiding storylines and point and target scoring mechanisms [124]. A target scoring mechanism used in an AR aircraft walkaround guidance system can be said to make use of experiential learning.

Other pedagogical factors were relatively rare in the studies examined. It is arguable and has been previously argued [83] that pedagogies not rooted in constructivism are rarely mentioned in XR education literature. In addition, many articles lacked sufficient details to determine the underlying learning theory employed or to be employed. Nonetheless, several studies have used experiential learning, situated learning, and connectivism. Experiential learning theorizes that learning is best achieved when the learner engages in and, importantly, reflects upon, direct experience [125]. Several of the examined articles that implemented a VR solution focused on the learner performing actions upon, and otherwise directly interacting with, various cockpit and aircraft system elements [88, 126]. These VR learning scenarios mirrored actual flight situations, thereby encouraging learning through experience; that is, experiential. The AR procedure trainers that seek to replace the non-digital training of procedures are, to an extent, creating a more experiential learning experience by allowing a more ‘hands-on’ but virtual experience [18, 56]. The related theory of situated learning acknowledges that instruction in settings removed from actual performance tends to fragment the capacity of the learner to execute the skill beyond the learning context [125], incorporating an oft-used simulation metric known as transfer of training (ToT).

Situated learning requires instruction to take place in appropriate spaces and be practical and hands-on wherever possible [127]. In terms of situated learning, the greatest enhancement to flight simulator training may result from the implementation of MR. MR allows the ability to view situations directly [128] or abstractly [129] in manners that are not viable with traditional technology. For example, pilot training using live and directly controlled drone footage was hybridized within a virtual environment designed for crew [128]. There are several emerging learning theories that more fully acknowledge the connected nature of learning [130], such as those necessary with the advent of education undertaken through XR [131] and other types of e-learning. Examples of learning theories that better address the networked technology of XR include expansive learning [132] and connectivism [131]. Several studies included aspects of expansive learning, although not sufficiently to be classified as such. However, connectivism was fully identifiable in several articles. Connectivism, as outlined by Scavarelli et al. [131] and largely based on Siemens [133], focuses on the occurrence of learning within a network and the connection of the learner to external nodes, such as smartphones. Knowledge of the function of cockpit controls and their location within the flight deck, held locally or in the cloud and connected to the student when sought, is arguably the mechanism of many MR or AR procedure trainer implementations [18, 85]. XR systems that make use of ML [97, 101], and even those that provide programmatic guidance [98], connect the learner to the knowledge of the procedure without the presence of an instructor and can be classified as connectivist. Another example involves connecting the learner and instructor using XR technology and allowing each to view the situation from a different perspective, as demonstrated in ref. [113]. The chronological progression of learning theories highlighted by Tracey [108] and the educational context of XR described by Engeström [132] may lead to the interpretation of many of the included articles classified as constructivist as being connectivist.

#### Intrinsic factors

In addition to the pedagogical factors motivating the adoption of XR technology for flight simulator pilot training, five ‘intrinsic’ factors were present in the articles examined: increased immersion, increased motivation, increased enjoyment, more personalization, and deeper learning (Fig. 5). These intrinsic factors largely coincide with those presented in two previous reviews of XR education [58, 134]. Increased immersion through XR, particularly through VR, was a motivating factor in 26 of the articles. Immersion may be described as the extent to which the operator feels present in the generated environment; in the context of an XR flight simulator, they are enveloped, in vision and surrounding depth, by the computer-generated environment of the cockpit (i.e., an independent environment), while physically located in another environment [135, 136]. Such immersion, as described by Lawrynczyk [137] based on the work of Slater and Wilbur [138], is dependent on the alignment of sensory modalities, enveloping the operator in a congruent, interactable, and sufficiently vivid digital environment. Although it is possible to achieve this type of immersion with MR and AR through the use of external physical environment queuing, such immersion primarily occurs in the domain of VR. VR attempts to isolate the operator from the surrounding environment, as outlined previously. Among the articles that emphasized increased immersion as an intrinsic factor in the use of XR, 85% used VR rather than other technologies on the spectrum.

Most studies that mentioned increased immersion as an inherent motivation for the adoption of XR neither assessed, quantified, nor directly researched how comparatively immersive XR was or why this was the case. Those articles that specifically addressed immersion took two approaches: (1) How immersive is XR compared with traditional simulators? and (2) Does increased immersion enable techniques that are otherwise impossible? The traditional simulators used for comparison with XR utilized desktop PCATD systems; more plainly, they used computer monitors. Livatino et al. [139] and Zhang [140] addressed immersion but did not evaluate differences in their investigations. Lawrynczyk [137] evaluated participants’ subjective immersion. The participants in this study noted “how immersed they felt in the simulated environment when they could turn their head around and still see the aircraft” [137]. Increased immersion, although in need of greater quantification in future research, can play a role in accomplishing effects that are not otherwise achievable with existing simulators. For example, coupling immersive flight simulations with galvanic vestibular stimulation can trigger vestibular flight illusions [141].

Several of the examined XR studies took for granted that the increase in immersion of a flight simulator necessarily increases user engagement [142, 143] without direct measurement. Immersion and engagement are not synonymous; a fully immersive experience can occur with negligible participant engagement, and* vice versa*. Given the difficulty of measuring immersion objectively, the likes of Bell et al. [92] focused on engagement for its own sake and effectively viewed it as only a proxy for immersion, whereas that assertion has arguably not yet been validated in the existing literature.

Deeper learning was an intrinsic factor of XR technology in 14 of the examined articles, tending to refer to learning that engages higher-level active cognitive processes [144]. As with the previously discussed pedagogical motivating factors, the examined articles tended to suggest that XR technologies enable deeper learning. The converse of deeper learning would be the various types of surface learning that use only lower-level cognitive functions, such as rote learning. In the context of flight training, using the levels of learning structure [109], deeper learning would be learning that takes place at the level of understanding, and particularly towards the level of correlation. Constructivist approaches enabled by XR technology in this application, as previously highlighted, as well as increased immersion, arguably reinforce intrinsically deeper learning. Enablers of these intrinsic factors include allowing the learner to explore [85], be immersed in the simulation [145], and infer understanding or knowledge from experience within the XR environment [98].

The attempt by many researchers to have the learner move immediately to the level of understanding or higher, while in the very early stages of training, has potentially serious limitations if such learning replaces traditional instruction rather than augmenting it. The traditional structure and practical learning theory of flight training acknowledge the need to acquire repeatable information at the fact level (i.e., through rote learning) [109]. Within the framework of the four levels of learning, it is not possible to move to the level of understanding or to higher levels if the learner has insufficient basic knowledge [146]. Attempts to enable deeper learning, particularly through exploration, as emphasized by constructivism and enabled by XR, may have negative training consequences. For example, given the occurrence in all simulators of differences between the simulation and aircraft, insufficient guidance (e.g., by the instructor or AI) can lead to the ‘construction’ of compensatory skills [42, 147]. It may be necessary for the learner to be guided through the training process, whether by humans or machines, with due consideration of the limitations of the simulator and needs of the learner.

The needs of the learner (i.e., the trainee pilot) may be considered from many perspectives, such as individual learning styles. In flight instruction, given the adherence to traditional intructivism, such needs are not necessarily a priority. However, with the affordances of XR technologies and the increasing power and availability of computers, it is now possible for simulator flight instructions to allow for greater consideration of learners. In the examined literature, this consideration has been coded as personalization. While personalization considers the learning style of the individual, it also considers the broader concept of conforming the experience to the learner rather than the converse [148]. Six articles included a specific form of personalization. Such personalization included the ability to moderate the pace of training [98], modify incentive structures to better cater to specific learning preferences [124], enable training outside of more formal settings [98], and complete or repeat lessons without the need for an instructor [56] (i.e., asynchronicity).

Access to limited resources was identified in the application themes and previously discussed in this review. This theme contributes towards the intrinsic personalization of XR, in that the resources of the flight simulator are more accessible, both temporally and financially, allowing for more practice of flying tasks. Furthermore, the dematerialization of the flight simulator, as discussed previously, allows greater access outside more formal training settings (i.e., enabling distance learning). Collectively, these characteristics may be interpreted as a continuation of the greater access that has been possible with PCATDs, owing to the increasing power and reduced cost of computers. Because the flight instructor is also a limited resource, partial or full replacement with AI guidance through XR hardware could serve a similar role in intrinsically allowing personalization of the learner’s training experience [56]. XR serves as a platform for gamification of flight training and allows for personalization through modification of incentive structures, including modification to assist entire groups [124]. As with access to limited resources, this is not limited strictly to XR. It is often necessary for a flight instructor to modify, and therefore personalize, student instructions to ensure learner progress. The ‘ingenuity’ of the flight instructor [149] being the enabler is often touted. The personalization of simulator flight training is part of what assists XR to increase motivation and enjoyment generally and intrinsically.

Six articles included increased motivation as an intrinsic factor in the use of XR, and three included increased enjoyment. Enjoyment, or relatively increased enjoyment, can contribute to motivation (e.g., task value), result from being motivated, or occur independently of motivation. Among the aviation articles examined, the prevalence of these factors (Fig. 5) was notably lower than that reported in a previous broader VR education review [134]. This may be linked to the underlying instructivist education philosophy of much of flight training. Increased motivation can originate from many sources, including the increased realism of the XR simulation [150] or the gamification of the simulation [98]. The increased motivation to use a flight simulator, which is sometimes referred to as motivational fidelity, may also play a role [151]. The increased realism of XR flight simulation can be attributed to or the use of simulator motion [45]. The specific mechanisms through which XR increases learners’ enjoyment of the training process can vary, although the increase in enjoyment is measurable [139]. Gamification can again play a role in increasing enjoyment [124], as can immersion [152]. Although not mentioned in any of the articles examined as a specific driver of increased enjoyment or motivation, Gartner’s hype cycle is relevant to the field of XR technologies [153]. The studies included in this review covered the period during which XR, and more particularly VR, followed the cycle expected of emerging technologies, including an extended period during which it was ‘over-hyped’ [154].

#### XR spectrum and technology used

##### Spectrum

**Fig. 6:**
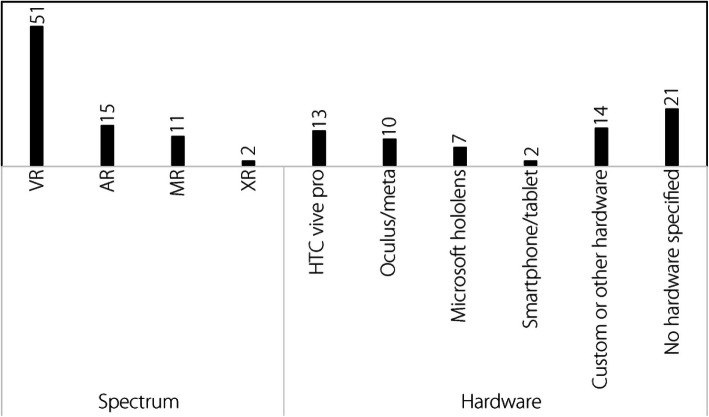
XR spectrum and technology

The types of XR technologies utilized in each study are shown in Fig. 6. Coding was based on previously specified definitions, except for XR. XR was used when the implemented technology was not clearly definable within the regions of the reality-virtuality spectrum otherwise covered by VR, MR, or AR. As with previous codes, it should be noted that the study may have utilized more than one technology. Approximately 85% of the studies used only one XR technology, whereas 15% used two or more technologies. As immersion is viewed as an important intrinsic factor in XR technology, it is perhaps not surprising that the majority of research would be on VR rather than other XR solutions. Individually, VR accounted for 65% (*n* = 51) of the technologies utilized, and although VR is still clearly the XR solution of greatest interest for flight simulators, AR is receiving increased interest: 70% of the studies that focused on AR were published in only the past three years.

The emergence of XR as both a consumer technology and a novelty to a larger population, compared with its long history in academic research, was only possible by the beginning of the twenty-first century owing to the increasing power and portability of computers. The novelty and ‘hype’ of the category was further intensified by the launch of consumer-grade hardware by major technology companies [155, 156].

##### Hardware

Figure 6 shows the most prevalent manufacturers specified; unfortunately, 45% of the articles did not specify the hardware used for the research (e.g., [118]) or in some cases, did not use any hardware (e.g., [96, 157, 158]). The HTC Vive Pro and Oculus/Meta varieties were the most common VR headsets used in the relevant studies, whereas Microsoft HoloLens was the most common AR device. Oculus/Meta includes all VR headsets manufactured by the company, including all generations of a particular headset. Microsoft Hololens includes all generations of the device. The use of the HTC Vive Pro and Oculus/Meta headsets, designed specifically for VR, accounted for 50% of all specified hardware. Notably, recent updates to the Meta headset software and SDK have enabled some VR devices to function as VST-HMDs, although this feature was not examined in any of the research articles. Given the intrinsic factors and specific requirements for VR, it is understandable that discrete hardware was the most common. However, given the wider prevalence of general-purpose handheld devices for AR [30], it is surprising that a discrete OST-HMD (i.e., Microsoft Hololens) was the most common AR hardware for flight simulator training research. However, AR is primarily used in the flight simulator training space to assist learners with in-cockpit checks [56]. Executing such checks generally requires “by hand” manipulation of instrumentation and controls by the pilot. That is, one hand is appropriated in holding a non-HMD device, which may limit the use of such general-purpose devices, at least for this type of training. Nevertheless, general-purpose devices such as smartphone/tablet were used in other AR training scenarios researched [98]. Microsoft Hololens was also the preferred individual hardware for MR research, although collectively Custom or Other Hardware was collectively used more often.

#### Domain and level of training

The selection of hardware for a particular training simulator can be based on many considerations, including the aviation domain and the intended level of training.

##### Domain

**Fig. 7:**
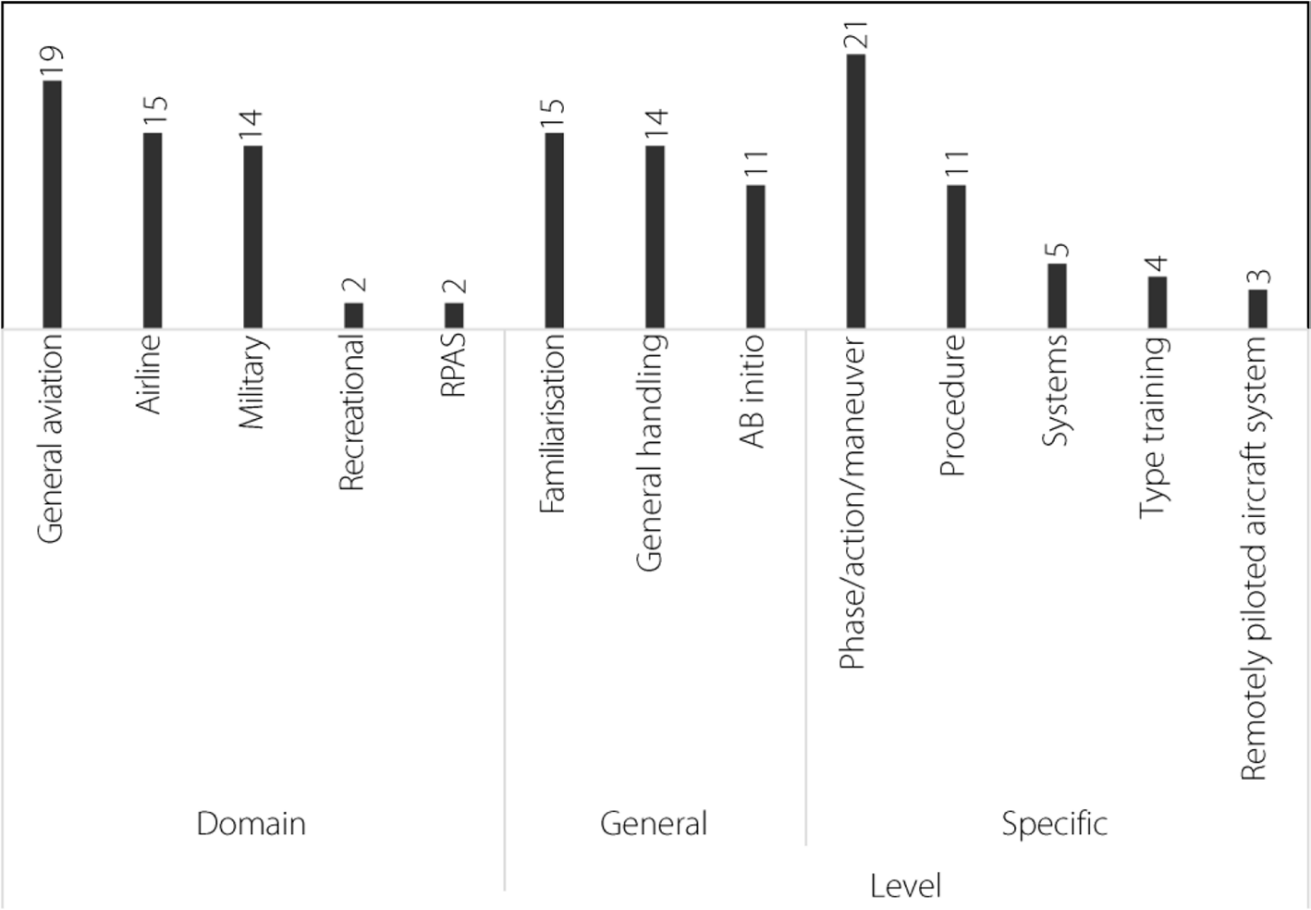
Domain (aviation) of training and level of training

The thematic codes for the various aviation domains using XR flight training simulators are largely based on the definitions used in Australian legislation and otherwise common in the legislation of other nations, and the ICAO. The exception to thematic codes matching the legislative framework is the recreational code, which in this analysis includes aviation activities that are not generally legislated directly by national civil aviation authorities, such as sailplanes. GA [159] was the most common aviation domain (Fig. 7). Although GA is not the only aviation domain in which flight training occurs, it represents the majority of flight hours [160]. Given this overrepresentation, it is understandable why articles on flight training, whether XR or not, were most often based on the GA domain. Applications within the GA domain would include skills and standards required for the initial issue of licenses and ratings up to and including the CPL and multi-engine command instrument rating. Such skills and standards would include NTSs, such as maintaining situational awareness [56, 126], common standards [161], rating standards [140, 162], and familiarization with light aircraft cockpits and systems [85, 98]. The dominance of XR solutions in the GA domain largely explains the significant number of studies (*n* = 16) that simulated the cockpit, systems, procedures, or structure of the Cessna 172 common trainer.

Both airlines and military aviation were almost equally represented in the research, although they were still less represented than GA. Airline activities would include heavy aircraft cockpit and procedure familiarization [161]. Military aviation activities overlap with many of the same skills and standards as other domains but are conducted within and for military purposes such as helicopter landings on a naval vessel [163]. In principle, simulators are utilized substantially across all three domains; hence, these studies have also been similarly reported. Training for sailplane pilots [89] and the use of a remotely piloted aircraft system (RPAS) [117] were also present, and included in the recreational and RPAS domains, respectively.

##### Levels of training

The training levels were subdivided into the General and Specific groups (Fig. 7). The General group includes familiarization, general handling, and ab initio. The ab initio code is effectively limited to the GA domain where such training usually occurs, whereas familiarization and general handling cover all five domains. Familiarization includes activities such as introducing the cockpit [98] or the often-mentioned but unfortunately never formally researched aircraft pre-flight walkaround. Ab initio includes all generic activities required for normal operations of the aircraft, where general handling is considered to be the refinement of maneuvers to the standard required for issuing a license. Starting the aircraft engine and landing the aircraft are examples of ab initio and general handling, respectively. The specific group contains more narrowly defined and definable training levels.

##### Specific skills

**Fig. 8:**
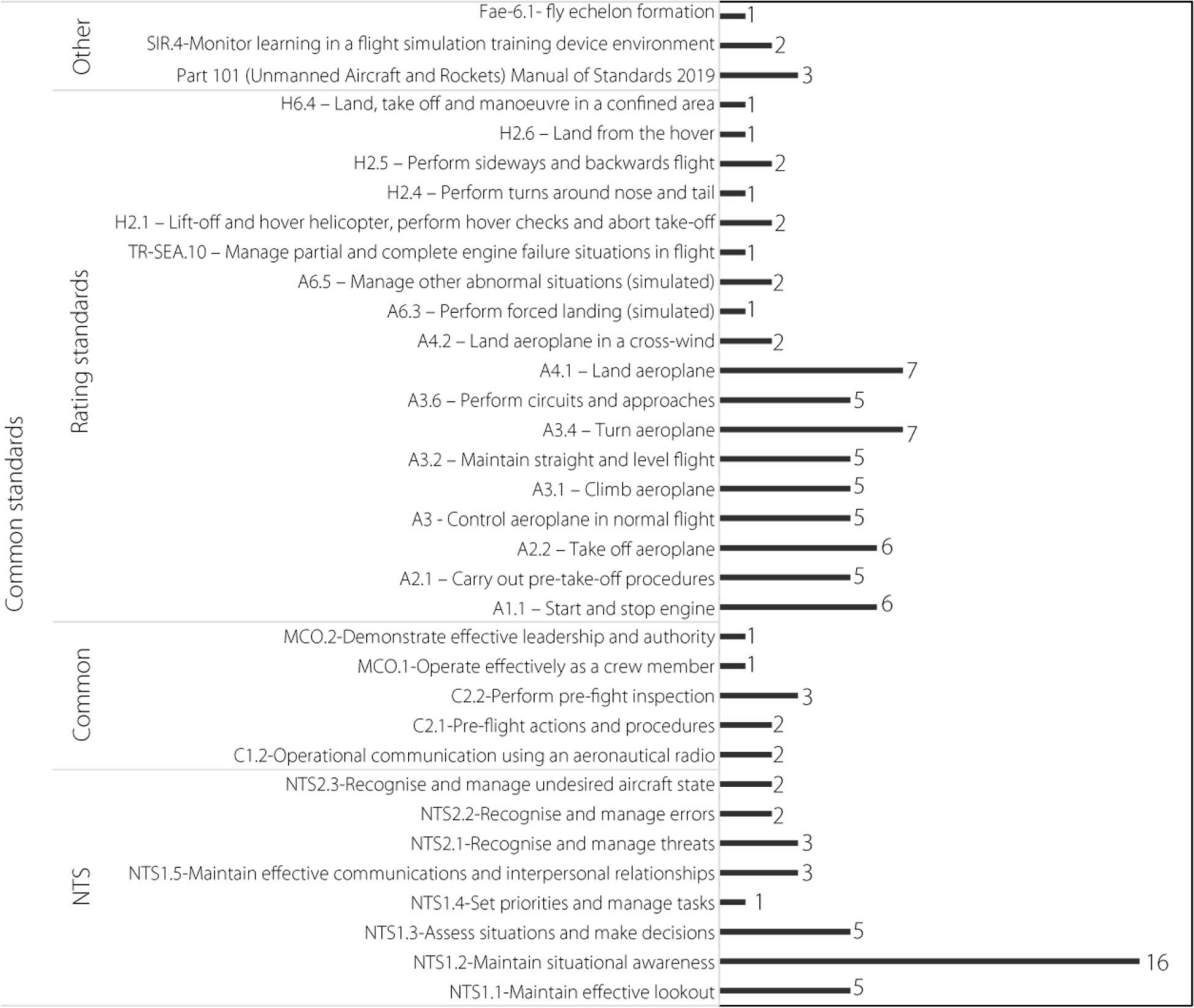
Thematic coding results for specific skills

During thematic coding, it became clear that the skills targeted by simulator training, with a few exceptions, aligned with the standards in ref. [41]. These codes were then used a priori. However, notably, none of the included articles directly specified these codes. The codes within the standards were divided into six groups based on English Language Proficiency and Flight Activity Endorsement Standards. Within these sections are collective unit competencies, such as Manage Fuel, which contain the individual skills (codes) required for that unit. Among the examined articles, codes from the Common Standards and Rating Standards groups were primarily identified (Fig. 8). Common Standards refer to the skills and knowledge required regardless of the aircraft or license type. The collective unit competencies within Common Standards can be grouped into NTSs, Common standards, and Manage flight crew during multi-crew operations. NTSs refer to internalized attributes and capabilities that are not directly related to the mechanical operations of the aircraft and its controls, but are necessary for the safe and efficient conduct of flight operations [164]. NTSs are considered equally important to technical skills, particularly in maintaining safety [165]. NTSs, along with human factors to a lesser degree, serve as measurable and reliable indicators of traditional airmanship [166]. The NTS most commonly identified in the included articles, was maintain situational awareness (NTS1.2). To maintain situational awareness requires systematic monitoring of the aircraft and its systems, including the collection of information necessary for the continuous management of systems, and the systematic monitoring of the flight environment, including the collection of information necessary for the revision of planned operations within that flight environment. Within the XR flight simulator, the teaching and assessment of how the learner can maintain situational awareness includes AR examples of systematic scan techniques required for monitoring aircraft system displays [56, 126] and full VR simulators that enable better monitoring of the flight environment [140]. These examples, as well as most others in the examined articles, are largely facilitated by a greater ease of immersion within flight and cockpit environments, supported by low-latency head movement tracking.

The second and third most common NTSs, as shown in Fig. 8, were maintain effective lookout and assess situations and make decisions. To maintain effective lookout involves (1) maintaining traffic separation through systematic visual scanning and interpreting that visual information for determining the traffic situation, (2) monitoring radio communications and interpreting those communications for determining the traffic situation, and (3) performing ‘airspace-cleared’ procedures prior to maneuvering [41]. In the examined articles, maintain effective lookout was largely restricted to the visual parts of the skill [114, 167]. Notably, even though such an enabling technology would allow far greater opportunities for visual see-and-avoid training during simulation, owing to the perceived ease of viewing through 360 degrees [87], this was only present in a single article [167]. To assess situations and make decisions involve undertaking a continuing process of problem identification, analysis, solution identification, enactment of selected solutions, and monitoring and re-evaluation of actions taken [41]. The assessments and decisions associated with maintaining separation between aircraft in close proximity [168], performing a circuit (aviation) [169], or simply flying the aircraft [89] are examples of assessing situations and making decisions in the XR flight simulator. This is linked both directly and indirectly to the other NTSs already mentioned, as well as to other NTSs and skills, such as those classified as rating standards.

Common standards include the more technical skills common to all aircraft and licenses that are necessary for the safe and efficient conduct of flight operations [164]. The most identified common standards skill was Operational communication using an aeronautical radio (C1.2), which was included in three articles (Fig. 8). Operational communication using an aeronautical radio (C1.2) is a skill that requires a person to be able to communicate in the aviation environment adequately, while using aviation English, through an aeronautical radio, for safely conducting flight operations [41]. This code has been represented in direct training applications such as communicating to obtain start clearance [161] or receiving operational information [170], and in research on secondary factors [143]. The second and third most frequently identified common standards were Pre-flight actions and procedures (C2.1) and Perform pre-flight inspection (C2.2), which were each used in two articles. The Pre-flight actions and procedures (C2.1) and Perform pre-flight inspection (C2.2) codes are both parts of the Perform pre- and post-flight actions and procedures (C2) unit. These two codes include administrative, review, and assessment activities that are required before conducting flight operations [41]. These activities include the crew-specific tasks conducted onboard [161] and the external ‘walkaround’ of the aircraft to identify defects or damage [94, 171]. Although common standards are requirements for all aircraft and licenses, these skills were the least common among the readily linkable skills taught in XR.

The rating standards (aircraft rating standards) codes [41] refer to the technical skills and knowledge required to operate specific categories of aircraft and are defined for each category of aircraft covered previously [172]. The rating standards cover such disparate skills as “considering the ground surface prior to attempting engine start” and the capability to perform a straight sideslip. Individual rating standards are generally more narrowly defined than NTSs. The most identified rating standards skill was land airplane, which was included in seven of the research articles (Fig. 8). Land airplane (A4.1) is an airplane category-specific skill that further stipulates the requirements for the correct execution of the maneuver, such as the requirement to control bounce after touchdown [41]. Given the disproportionate number of accidents and incidents during the landing phase [173], particularly within the flight training space [3, 174], the focus on the Land airplane (A4.1) skill and related Landing in a cross-wind (A4.2) is understandable. Despite the importance of landing, Control aircraft in normal flight (A3), which includes Maintain straight and level flight and Turn airplane, was the most identified unit of rating standards. As most flight time (57%) is in the cruise phase [175], it is understandable that the skills required for this phase of flight would collectively receive the most focus. Helicopter skills were far less often the focus of the training undertaken, or to be undertaken, in XR. This largely matches the patterns of fixed-wing aircraft and fixed-wing training flight hours and significantly exceeds those of rotary wings [176].

#### Limitations of XR

**Fig. 9:**
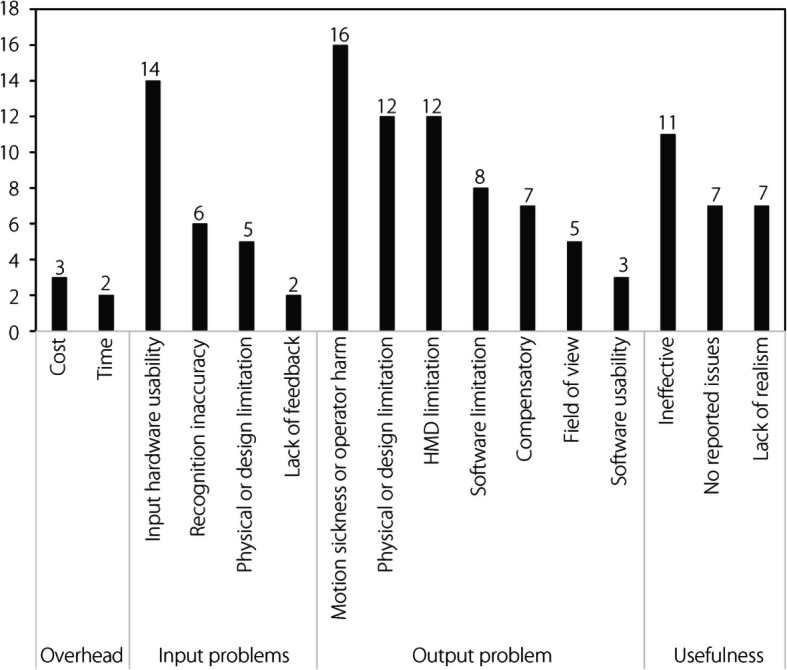
Prevalence for XR limitations

Flight training in an XR flight simulator, whether for the skills outlined or other skills, must be considered alongside various factors that can or potentially should limit the use of such applications. The limitations identified in the examined articles are largely consistent with those found in previous reviews of XR education [58, 134]. These limitations, shown in Fig. 9, include those determined by the implication of the findings in articles and those explicitly stated, whether derived from objective measures or subjective means. Notably, 23 of the 67 research articles did not discuss the limitations of XR despite the general need for academic balance in such articles. The limitations of XR flight simulators can be broadly classified into four categories: overhead, input problems, output problems, and usefulness.

##### Overhead

As with the implementation of any new technology or method, there are temporal and financial overheads. The cost overhead was noted in three of the examined articles, and the overhead in time was noted in two articles. The cost overhead of an XR simulator usually results from the need to acquire or create the hardware required for access to virtuality [142]. These upfront capital costs, although far lower than those associated with FFSs, must be considered in a broader context. For example, the cost per unit must be considered, given that the use of the technology is an enabler of distance learning and that there is a need for multiple units to create a shared space, particularly towards the virtuality end of the spectrum. XR is sometimes viewed as simply imposing an additional cost on trainees [177]. Despite the lower cost of a discrete HMD compared to an FFS, these are ultimately additional costs to be added to a PCATD. It is also unclear whether existing PCATDs can be readily upgraded by simply adding a headset, given requirements such as the graphics cards of many such headsets [178]. Some of these costs may be reduced by the use of personal handheld devices, but there will still be development and deployment costs. There is no guarantee that the overhead in terms of time will be reduced using this method. For example, there is still a temporal burden in the development and setup of XR software and hardware [142], as well as in becoming familiar with the new system [179]. Given the focus of many articles on applications in GA, the noted weaknesses of this sector [180, 181], and the general vulnerability of the entire industry [182], temporal and financial overheads have become critical considerations in the adoption of XR, requiring a nuanced assessment of the viabilities and limitations associated with such resource commitments.

##### Input problems

The input problems of XR flight training simulators are largely derived from the limitations of the specialized inputs that devices require for accessing XR, that is, the primary and secondary inputs. The input hardware usability was the most common input limitation, followed by recognition inaccuracy (Fig. 9). The primary input hardware, defined as the sensors that enable accurate real-time tracking of the free movement of the user/device in 3D real space, is largely limited by recognition inaccuracy. For example, the loss of VR HMD tracking is caused by roll, pitch, or yaw sensor inaccuracies [24]. Secondary input hardware, which includes sensors and devices that facilitate interaction (e.g., hand controllers or hand tracking), suffers from limitations in recognition and usability. For secondary hardware, issues of recognition and usability are sometimes difficult to distinguish. For example, within a particular XR implementation, the interaction with cockpit elements is a primary issue [4, 161]. Nevertheless, there are examples of sensor hand tracking [56] and controller tracking [113] that have usability limitations without a particular recognition limitation. General-purpose handheld devices and their associated touchscreens, which are the primary and secondary input hardware, respectively, do not have recognition or usability limitations for AR applications [85, 98]. Secondary input hardware in the form of tracked controllers and flight-specific inputs was most prevalent in the lack of feedback and physical or design limitations. The implementation of an XR simulator, in which the “hands on throttle-and-stick” is the only flight hardware, can result in a lack of feedback [24] or physical or design limitation when a controller imposes a physical burden owing to weight [4]. The subjective evaluation of the limitations of XR simulation must be considered in the context of flight training, specifically the evaluator’s role in such training. For example, student, instructor, and experienced instructor pilots may perceive the same system as having variations in perceived limitations [113].

##### Output problems

The output problems of XR flight training simulators were mostly a function of the output hardware limitations and were generally more numerous and common than other limitations. These XR primary output hardware, as already noted, are usually HMDs and handheld electronic devices, such as mobile phones. The most common output problem in the examined articles and the most common limitation overall was Motion Sickness or Operator Harm. Motion Sickness or Operator Harm includes issues of motion sickness, cybersickness, and physical harm to the operator, such as fatigue of the neck or spine. Motion sickness and cybersickness, which are similar phenomena with an inexact link [183], manifest as symptoms such as nausea, headache, and dizziness experienced by users, often from disconnects between learners’ visual cues and the vestibular or balance system. The part of the code (i.e., Motion Sickness or Operator Harm) addressing operator harm primarily pertains to the physical consequences encompassing fatigue and strain that arise from prolonged use owing to the mass of equipment on the head, neck, and spine. The vestibular parts of the Motion Sickness or Operator Harm code, as well as other output problems, are strongly interrelated. For example, the mass of an HMD, which is a Physical or design limitation, may result in spinal problems and simulator sickness during long exposures, according to Stanney et al. [184]. However, the significant facial discomfort resulting from the extended use of a particular HMD [87], which is a form of harm caused to the operator owing to design limitations, does not necessarily result in motion sickness. As with other output problems (e.g., software limitation) [87] and other aspects of simulator fidelity [185], motion sickness can be moderated by flight experience [24]. Insofar as flight simulators are used at every level of flight training, the specifics of the simulator, or in this case, the XR simulator, should perhaps be tailored to the trainee [186]. XR flight simulators that use some form of HMD (Fig. 6) must also consider the output problems caused by a lack of experience with HMD operations in their flight operations [161], such as for most civilian pilots.

Although a lower-represented output problem, the compensatory skills code is arguably the most critical. Compensatory skills are abilities developed by the pilot to perform the required tasks in response to limitations and discrepancies of the flight simulator [42]. While compensating in this manner in the simulator, the pilot can be said to be in “simulator pilot mode” [185]. When completely limited to the flight simulator, such skills may be of little consequence or even useful or appropriate [187]. However, regardless of which dimension of fidelity is incorrect, discrepancies between the flight simulator and real-world flight can become dangerous if such skills are inadequate or counterproductive during actual flight operations [42]. The development of compensatory skills was highlighted in seven of the examined articles. These compensatory skills were largely a result of the HMD screen resolution or constructions (i.e., HMD limitation or Software limitation) or the field of view (FoV) of the HMD, whereby the instruments or other elements of the simulated environment were not sufficiently clear. Such clarity limitations, whether due to the resolution of the screen, visual artifacts caused by the physical construction of the screen, or software rendering of a given element, mean that the pilots will create compensation strategies in an attempt to maintain situational awareness. An example is the screen door effect, which can occur owing to an inability to resolve the gaps between the pixels, resulting in a mesh-like artifact, resembling a “screen door,” which affects the design of screens for VR applications [188]. Such visual artifacts may require the pilot to move far more than in a real aircraft to read the indication on an instrument, as though they are near-sighted [24].

The FoV of an HMD was highlighted as an output problem in five of the examined articles, including in combat training [189]; however, it was not specifically emphasized as a cause of compensatory skills or Motion Sickness or Operator Harm. Previous research by Dixon et al. [190], suggested that an inadequate FoV in traditional simulators could result in the development of negative compensatory skills. Compensatory skills need not simply be a result of a particular output problem, but may result in more complex interactions of factors. Based on the work of Kennedy and Fowlkes [191], Lawrynczyk [137] suggests that trainees may alter their behavior to reduce the symptoms of motion sickness or cybersickness. Thus, by altering their behavior(s), the training received for a given real-world flight scenario may be rendered ineffective or negative to a certain extent. The development of compensatory skills or sickness in the trainee, or the causing of harm to the trainee, may prompt or necessitate limiting the use of XR flight training simulators.

##### Usefulness

The usefulness grouping in the thematic coding concerns qualitative research on user satisfaction or dissatisfaction, rather than quantitative efficacy, as addressed later in the meta-analysis. The more generic usefulness problems of XR flight training simulators were related to the lack of realism and perceived effectiveness (i.e., ineffective) of such systems. The realism or lack of realism that serves as a proxy for both objective fidelity and perceptual fidelity in this case refers to “the degree to which the trainee subjectively perceives the simulator to reproduce its real-life counterpart aircraft, inflight, in the operational task situation” [185] and the engineered and calculated replications of real elements. A lack of realism may result in or contribute to an ineffective simulator in terms of ineffective training. Seven of the examined articles found a lack of realism and 11 found the technology to be perceived as ineffective in simulator pilot training. The causes of a lack of realism included the mechanisms for interaction (i.e., input problems), presentation [87], and lack of completeness of the environment (i.e., output problems) [113], as well as questions regarding the interactions of XR technology with other flight simulator fidelity moderation mechanisms [91]. The causes of a lack of realism notwithstanding, the methods of evaluation were both pure opinion (i.e., simple questions) and a more formalized assessment, as discussed later in this review. The ineffectiveness of the XR flight simulators was evaluated based on user opinions, subjective assessment tools, and objective training evaluations.

Opinions on XR being ineffective were from both experts and novices, and these opinions were obtained in cases with (i.e., [113]) and without (i.e., [171]) exposure to an XR simulator. The objective assessment of the efficacy of these simulators or their lack thereof was largely limited to comparisons of flight performance or internal XR performance metrics. The time required to perform a flight deck task was a common metric that was generally unfavorable for XR compared with more traditional simulator technologies [4, 87]. Articles that evaluated objective flight performance criteria (e.g., altitude holding) showed varying qualitative effectiveness of XR simulators, including a rating of ineffectiveness under certain circumstances [137]. The methods of evaluating flight performance criteria were often inconsistent with the assessment standards commonly used in real-world pilot training. XR flight simulators may be rendered ineffective or unusable for certain types of training if certain combinations of limitations lead to “problematic levels” of simulator sickness. The development of compensatory skills during simulator flight training, including their development in an XR simulator, was not assessed in any of the examined articles; however, it has the potential to render such a system ineffective. Further, the transfer effectiveness ratio, which calculates the efficacy of a flight simulator “by measuring the aircraft training time saved by simulator use” [42], was not computed for any of the examined articles.

#### Subjective assessment of XR

**Fig. 10:**
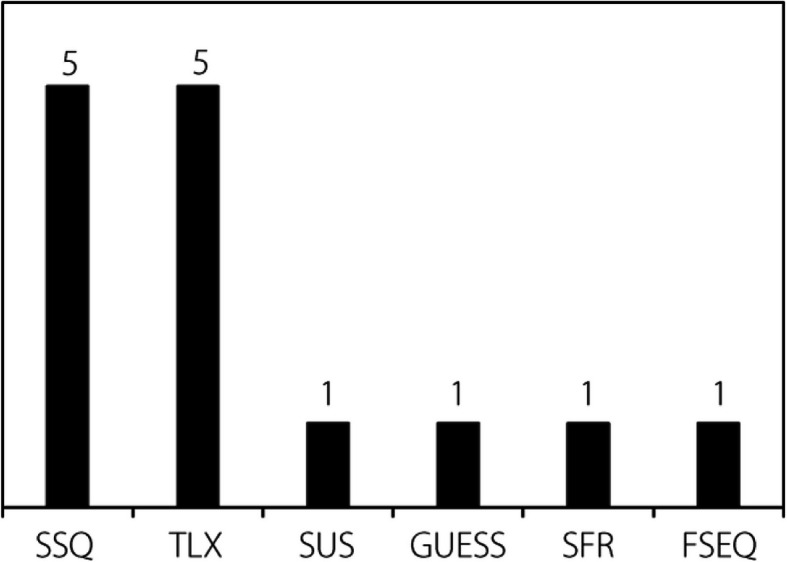
Frequency of subjective assessment tools

A subjective assessment of XR flight training simulators using more formal tools was performed in only nine of the studies. Gathering the required demographic data was not counted in either category. For those that included subjective assessment tools, the NASA task loading index (TLX) [192] (refs. [4, 90, 117, 140, 152]) and the Simulator Sickness Questionnaire (SSQ) [193] (refs. [4, 24, 140, 141, 152]) were the most common. Notably, the VR Sickness Questionnaire, which is a modification of the SSQ that is tailored for use in XR applications [194], was not a feature of any of the included articles. The TLX measures the user workload in the activity and circumstances, while the SSQ measures the physiological response to the technology. Additional tools that were identified were the Simulator Fidelity Rating Scale [195] (ref. [91]), System Usability Scale [196] (ref. [152]), Game User Experience Satisfaction Scale [152], and Flight Simulator Experience Questionnaire [197]. These additional tools (i.e., not the TLX or SSQ) can be collectively categorized as assessing the experience of using a simulator. The distribution of the subjective assessment tools across workload, sickness, and experience in the research was almost equally split (Fig. 10). The use of these types of subjective assessments may indicate anticipated issues with XR technology.

### Meta-analysis

#### Objective

Effect sizes for the meta-analysis were derived from five studies [4, 14, 140, 150, 161] that reported suitable objective flight performance data, following the outlined methodology. The results of the omnibus test of the model coefficients (*Q* = 5.054, *df* = 1, *P* = 0.025), which tests the null hypothesis that all predictors are unrelated to the effect sizes, were significant; the null hypothesis was rejected. Furthermore, the results of the residual heterogeneity test (*Q* = 1.508, *df* = 4, *P* = 0.825) implied no substantial heterogeneity beyond chance. The result of testing for the percentage of variability in the effect sizes that was not caused by sampling error (*I*^*2*^**~** 0.0%), although not a formal test, further suggests a lack of heterogeneity between the studies. The statistically significant omnibus test of the model coefficients, in conjunction with the non-significant test of residual heterogeneity, indicated a consistent intervention effect across studies. The overall meta-analytic estimate of effect size was 0.884 (*z* = 2.248, *P* = 0.025), which was interpreted as large [77]. The positive and statistically significant effect size indicated that the XR interventions assessed in the studies had a meaningful impact on the measured outcomes compared with the control.

**Fig. 11:**
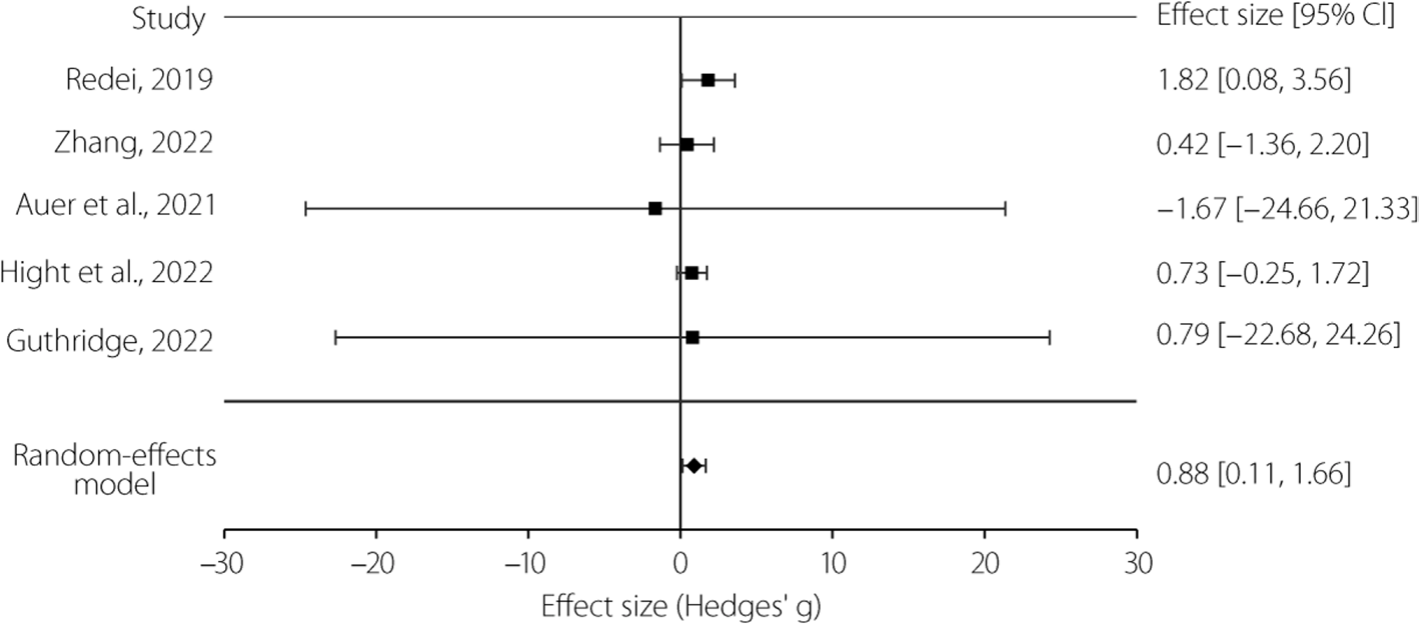
Funnel plot of effect sizes (objective) with 95%CI

The funnel plot in Fig. 11 shows the effect sizes determined for each study with 95%CIs. The overall meta-analytic estimate of the effect size (*g* = 0.884) is indicated by the diamond-shaped mark at the bottom of the figure. The meta-analytic estimate of effect size fell within the 95% confidence level of all included studies, and the non-significant test of residual heterogeneity supported the underlying assumption that different studies would estimate different yet highly related effects. The consistency of these intervention effects aligns with the essential methodological assumptions of the analysis and the use of random-effects meta-analysis. Notwithstanding, given the inclusion of effect sizes and variances that resulted for composites of multi-endpoint measures, further and more comprehensive research is warranted; this is not surprising given only five research studies to draw from.

#### Subjective

Three articles contained suitable subjective or non-flight data for the meta-analysis using the selected method [14, 140, 167]. These separately handled effects resulted in a non-significant omnibus test of the model coefficients (*Q* = 0.095, *df* = 1, *P* = 0.758) and a non-significant test of residual heterogeneity (*Q* = 0.721, *df* = 2, *P* = 0.697). Therefore, the predictors were not significantly related to the effect size, and there was no excess variability across the included studies. The overall meta-analytic estimate of the effect size of −0.055 (*z* = −0.308, *P* = 0.758) suggests a negligible and non-significant effect. The XR interventions assessed in these studies did not have a meaningful impact on the measured subjective outcomes compared with the control groups without XR. The disparity between the objective measures of XR efficacy across five studies was significant and positive, while the subjective measures of XR efficacy across three studies were insignificant, highlighting the need for objective and applicable measures in such research.

## Discussion

The potential of XR spectrum technologies as replacements for or augmentations to existing flight simulators is not easily interpreted from the present review. When considering the use of these technologies, a comparison with both traditional simulators and aircraft must be made, and evidence in favor of XR technologies is still emerging and remains limited. In examining the current literature, there is significant promise, but there are also substantial research gaps, particularly in terms of methodological and design weaknesses, and often unclear pedagogy, which affects the reliability of existing findings. While there is an overarching preference in the literature for constructivist educational theory, the explicit and direct application of such a theory and other educational theories remains limited. However, this review provides clear guidance for future research on the intended use of VR in specific aviation domains and training contexts, which should be considered by researchers in this field, albeit with a need to assess all pilot skills more comprehensively. Furthermore, while there is acknowledgment of the risks associated with the development of simulator sickness and compensatory skills consonant with traditional simulators, these and other concerns are inadequately addressed. Although the meta-analysis of this review shows improvements in objective flight performance outcomes, it also strongly suggests a need for further research to verify the benefits of these technologies and explore secondary outcomes. The increased research and, by proxy, preference for XR flight simulators in recent years, with the likelihood of continuing growth (Fig. 4), is unequivocal from the present review.

The results of the meta-analysis of this review, as with much of the remainder, must be interpreted with care. Although the overall meta-analytical effect size (*g* = 0.884) indicates a statistically significant and large positive effect of the intervention from XR technologies, this finding must be considered cautiously. This positive effect size suggests that XR interventions, primarily VR, enhance specific skills and tasks required by pilots (objective performance measures). However, it does not appear to improve the subjective measures to the same overall effect. This may indicate the potential for procedural improvements without corresponding improvements in pilot techniques [198]. The lack of heterogeneity in the meta-analysis results suggest a relatively consistent intervention effect. However, this may be misleading because of the methodological limitations of the included studies and the paucity of such studies. The limitations of some of the included studies, such as small sample sizes and potentially limited designs, could conceivably have resulted in an overestimation of the effectiveness of XR interventions. Therefore, while XR shows promise, it is necessary to conclude that additional research is required to validate intervention effectiveness. It is also notable and potentially concerning that, of the included 67 articles, only five (7.5%) contained adequate objective data for inclusion in the meta-analysis. Given the widespread intention to adopt this technology, including those expressed in the articles examined in this review, the lack of substantive research on the effectiveness of XR simulators contrasts sharply with the literature on traditional simulators. This weakness has been acknowledged in a few studies [4, 90, 152].

The literature clearly indicates that XR spectrum technologies, particularly VR flight simulators, have several inherent strengths and weaknesses in this specific application. The immersiveness, cost-effectiveness, and portability of these technologies surpass those of older technologies that they seek to replace. Regarding the enhancement of piloting skills, particularly in emergency procedures and spatial awareness, researchers view the immersive and accessible aspects of XR technology as likely to improve training effectiveness. Although the existing literature generally only addresses these aspects conceptually, these technologies may also support distance learning and broaden the scope of aviation education. These strengths must be considered alongside existing weaknesses. Such weaknesses include the limitations of the technology itself, as well as issues related to its usage context and limitations in the current research landscape. These limitations include inadequate research on the propensity for simulator sickness and the development of compensatory skills, both independently and as a consequence of simulator sickness. Although compensatory skills are a risk for all flight simulators, the risk of developing such skills in response to simulator sickness must be carefully considered in future research. Furthermore, the literature included in this review, including studies that attempted to quantify the training effectiveness of XR flight simulators, generally lacks specific learning strategies based on established educational theory. This contributes to the methodological weaknesses of these studies and may limit the effectiveness of XR flight simulators in their application.

The learning approach for XR flight training simulators that is preferred by researchers is one of the clearest conclusions that can be drawn from this review. While this approach is strongly implied, as shown in the thematic analysis, most research articles lack an adequate foundation in established educational theory. The lack of grounding in either modern educational theory (e.g., constructivism) or the older approach preferred by industry (i.e., instructivism) may misrepresent the capabilities of the technologies by limiting their effective application. Constructivism is the dominant theory used in the literature, with no particular consideration of the flight training industry’s preference for instructivism in pilot training. Despite this preference for constructivism, there is a near-complete absence of a direct connection with educational theory. This theory has largely been inferred by the reviewers rather than explicitly stated in the articles. An example of this lack of theoretical underpinning is the justification for the use of VR based on its inherent capacity to provide engaging and interactive learning environments. The theoretical underpinnings that would support this approach, such as constructivism as a learning theory, have not been addressed. Given the intention to adopt these technologies in real-world applications, including within industry, this general lack of educational theory and a complete curriculum is of concern.

GA is the primary domain in which these technologies are likely to be applied. Given the possible perceived cost reductions compared with traditional simulators and the savings in flight hours, the focus on GA is understandable, which naturally influences the level of training that has been researched. General handling of the aircraft, familiarization, and ab initio level training are all targets in the examined literature. The skills to be trained showed clear patterns linked to the perceived immersiveness and spatial awareness achievable using XR. The maintenance of situational awareness (NTS 1.2) is by far the skill that receives the greatest focus. The concepts of assessing situational awareness and maintaining an effective lookout are also strongly featured. Several ab initio skills, including the maintenance of straight and level flight and the turning of airplanes, are highlighted. The significant use of traditional simulators for training instrument flying skills is not reflected in the literature on XR flight training simulators. Although this has not been addressed, it may be linked to the lack of a need for the benefits of XR (e.g., the ability to look around the environment). The current focus of research in these domains, levels, and skills, when considered alongside the other findings of this review, indicates clear areas for future researchers to prioritize, such as more complete skills coverage and attention to the full curricular context.

The most important recommendations for those seeking to conduct research in this field concern the method, structure, and direct and indirect effects on those subjected to XR interventions. Constraints on available subjects, the extent of educational deviation of those subjects, monetary and temporal limitations, and existing institutional (e.g., flight school) structures often limit the research viability. It may be necessary for researchers to consider the use of quasi-experimental methods and address internal and external sources of invalidity to the extent that the situation allows. However, it is certainly necessary for those intending to conduct such research to select more suitable, or in many instances any, research design. The adoption of the approaches used in the traditional flight simulator literature, such as ToT, would be of similar benefit in proving these technologies. The difference in approaches between academia and industry is a point that researchers ought to consider. The flight training industry, for better or worse, works within a highly structured educational environment and makes use of long-established training approaches. It would be valuable to ground XR simulator training research in the accepted structures and techniques of flight training. For example, if an Australian researcher wishes to examine the effect of increased spatial awareness within a VR HMD on downwind tracking, the researcher is attempting to train the learner to the NTS1.2, NTS1.3, and A3.2 skill codes [41]. Such grounding would (1) enable more direct comparisons between studies, (2) support the uptake of the technologies by fundamentally addressing perceived difficulties in incorporating them into recognized training syllabuses [199], and (3) be of great value to non-academic readers (e.g., flight instructors).

## Conclusions

This review sought to explore the effectiveness of XR technologies across the spectrum as an adjunct to or full replacement for traditional simulators in pilot flight training, and to identify the motivating factors for its use. This systematic review was conducted using the QUOROM research framework adapted for educational studies. The existing literature suggests that XR flight simulators have the potential to be effective training tools for pilots, and the motivating factors for their development have been identified. XR simulators appear to offer a safe and cost-effective means of training pilots, including highly specialized training scenarios. However, multiple serious limitations exist in the research, such as the potentially higher occurrence of simulator sickness and cybersickness compared with more traditional flight simulators. The effectiveness of XR technologies for pilot training and learning has yet to be fully established through robust research trials that examine ToT. Substantial research on all variants of the XR flight simulator is still required to establish whether they are truly capable of augmenting or fully replacing traditional flight simulators. Despite the lack of proper educational and research designs in most of the reviewed studies, there is significant motivation to conduct such research. This novel systematic review and meta-analysis represents a significant attempt to shape and direct better research on the rampant technological XR development in a time of increasing pilot shortages and aviation growth.

## Supplementary Information

Supplementary Material 1. Appendix A.

Supplementary Material 2. Appendix B.

Supplementary Material 3. Appendix C.

Supplementary Material 4. Appendix D.

## Data Availability

The datasets presented in this article are not readily available because of restrictions on their distribution to parties external to the original research, as imposed by the ethics approval. Requests to access the datasets should be directed in the first instance to the corresponding author.

## Abbreviations

ATC: Air traffic control
AR: Augmented reality
COTS: Commercial off-the-shelf
CPL: Commercial pilot license
CONSORT: Consolidated standards of reporting trials
FFS: Full flight simulator
FoV: Field of view
GA: General aviation
HMD: Head-mounted display
ML: Machine learning
MR: Mixed reality
NTS: Non-technical skill
OST-HMD: Optical see-through head-mounted display
PCATD: Personal computer aviation training device
QUOROM: Quality of reporting of meta-analyses
RCT: Randomized controlled trial
RPAS: Remotely piloted aircraft system
ToT: Transfer of training
VST-HMD: Video see-through head-mounted display
VR: Virtual reality
XR: Extended reality
3D: Three-dimensional
TLX: Task loading index
SSQ: Simulator Sickness Questionnaire
