# Supplementary material for: Applications of extended reality in pilot flight simulator training: a systematic review with meta-analysis
Source: Vis Comput Ind Biomed Art. 2025 Oct 23;8:25. doi: 10.1186/s42492-025-00206-w (PMC12546163; doi:10.1186/s42492-025-00206-w)
Supplement: Supplementary file 1 — Supplementary Material 1. Appendix A. [file 42492_2025_206_MOESM1_ESM.pdf]

Appendix A – Description and Primary Outcome of the 67 Included Articles

Paper	*Year*	*Description*	*Outcomes or Findings*	*xR*
Mowafy and Thurman	*1993*	*Evaluation of a VR tool for training through debrief, based upon previous simulated flight.*	*Some indication of differences in performance, suggesting that 3D stereoscopic systems be the most effective trainers for building mental models of three-dimensional spatial relationships.*	*VR*
Vince	*1993*	*Discussion of the overlap and development needs from traditional to VR simulators.*	*As at the time of public head-tracking sampling rate issues were noted, motion sickness problems were noted, and high time and labour costs were noted.*	*VR*
Amburn and Marshak	*1996*	*Examination of a HMD (VR) system for flight replay, for training purpose, and the situation awareness implications.*	*SA measured using probe questions. Theory of signal detection (TSD). Subjective preference for traditional display.*	*VR*
Magee	*2000*	*Describes the technical and human issues of the development of a VR simulation for the training of landing skills, as well as a proposed experimental plan.*	*The visual and motion cueing present a significant technical challenge for the development of a VR sim for training purposes.*	*VR*
Bles and Wertheim	*2001*	*Review of simulator motion sickness, the specific implications for HMDs, and possible training implications*	*The correct use of virtual environments, particularly through visual and auditory cues, can minimise motion sickness*	*VR*
Doerr, Schiefele	*2001*	*Explores the various application of VR in the training of pilots, including in-cockpit MR for systems education*	*Immersive virtual simulation, and specifically a virtual cockpit, enhances the training standard of classroom learning and CBT. Tracker and system lag were identified as serious limitation of the implementation. The FOV of the HMD used was identified as a limitation, though the availability of HMDs with greater FOVs is acknowledged.*	*VR, MR*
Aka and Frasson	*2002*	*Focussed on the development of software to enable distance*	*The developed system, which uses VR, provides*	*VR*

		learning, augmented with AI, for VR flight simulator training.	*automated real-time assistance.*	
Ntuen, Adams	*2004*	*Comparison of training performance between IVE (immersive virtual environment) in the form of a HMD, and a non-immersive, showing variation between measured tasks.*	*The results indicate that NIVE may be good for training GA related altitude tasks while IVE may be appropriate for vertical airspeed related tasks.*	*VR*
Bauer and Klingauf	*2008*	*Documents pilot responses to the implementation of a VR procedure trainer, for replacement of CBT.*	*VR is accepted readily in pilot training. A VR procedure trainer can is a better preparatory procedure trainer than is traditional CBT.*	*VR*
Gu, Wu	*2009*	*Development of a xR flight training simulator, with focus on open architecture and minimisation of cost.*	*A MR flight simulator that uses a semi-virtual cockpit, suitable for training, was developed.*	*MR*
Sciarini, Elfe	*2015*	*Evaluation of a training simulator using CastAR glasses, by qualified military pilots, and a subsequent survey. Training aspect primarily about proficiency training.*	*The prototype was well regarded by Naval Aviation SMEs. The system was found to be a viable visual interface, and a suitable task rehearsal tool.*	*AR*
Cardenas, Letdara	*2017*	*Development of a creative MR/AR training simulator, using modern RPAS and communication architecture to increase immersion*	*Proposal for an xR flight training system with increased immersion for the bridging of the gap between virtual and reality.*	*MR, XR*
Brown	*2017*	*Presentation on possible XR applications in aviation, noting the possible teaching of flows.*	*Negligible.*	*VR, AR, MR*
Montalvo and Pihl	*2018*	*Presents a methodology for the designing of an AR view map for improving spatial perception in a flight training simulator.*	*An 3D AR view map was found to be more intuitive and user-friendly than traditional map views.*	*AR*
Kazenmaier, Gerboni	*2018*	*Design of a rotary-wing simulator, with evaluation of training validity by pilot-in-the-loop experiment, including fidelity rating.*	*The developed VR helicopter suitable for training, validated through pilot-in-the-loop experiments*	*VR*
Lawrynczyk	*2018*	*Compares cognitive load, user experience, and performance metrics during flight training*	*There was no significant difference between the traditional flight simulator and the VR*	*VR*

		between BADS (broad angle display system) and VR graphics	*version, in terms of user experience, cognitive workload, and performance.*	
Brown	*2018*	*Presentation on the use of MR & AR technology in aviation, including simulators for pilot training*	*Negligible.*	*AR, MR*
Giaimo	*2018*	*Press release on the adoption of VR flight training simulators for pilot instructor training in the United States Air Force.*	*Negligible.*	*VR*
Gustafsson	*2018*	*Comparative evaluation of VR (commercial off-the-shelf) and 2D (monitor) flight training simulator, evaluated with questionnaire and SSQ.*	*The VR HMDs available at the time of publication did not meet the technical requirements of a flight simulator, based upon objective technical comparison. Possible evidence of the development of compensatory skills among trainees. Development of simulator sickness in VR, based upon SSQs during the simulation, is highly personal.*	*VR*
Biddle and Buck	*2019*	*Report by Boeing personnel of the in-house development of a VR training simulator for CRM training*	*The use of VR and AR interfaces, when combined with adaptive training technologies, can be used to offload certain training from traditional simulators.*	*VR*
Redei	*2019*	*Development and learning evaluation of a VR flight simulator, making use of a motion system.*	*A VR flight simulator, which made use of gesture-tracking, improved learning, and training outcomes.*	*VR*
Brown	*2019*	*Poster outlining possible use of MR, particularly AR, for training of pilots in the recent generation*	*Negligible.*	*AR*
Pennington, Hafer	*2019*	*Outlines quasi-experimental ToT research, examining the efficacy of VR flight training with vary levels of structure.*	*Strictly structured training using the VR simulator showed accelerated positive transfer to the real aircraft.*	*VR*
Mendonca, Keller	*2019*	*Poster proposing research on a VR training solution for helping pilots*	*Proposal for research using VR to train pilots for birdstrikes*	*VR*

		reduce the bird strike problem		
Ommerli, Mirzaagha	*2019*	*Examination of the direct and indirect effects of fluency, presence, and interactivity in VR on prospective memory and working memory.*	*Greater interactivity and presence were correlated with better prospective memory.*	*VR*
Bell, Kelsey	*2020*	*Outlines the development of a ML model for the detection of lapsed engagement during simulator training, using the additional data available from a VR HMD.*	*Negligible.*	*VR*
Salazar and Mario Sergio	*2020*	*Near identical content to [89]*	*Development of an AR mobile application for the training of a pilot for a new panel layout, based on the C150.*	*AR*
Fussell	*2020*	*Examines the intentions of students enrolled at part 141 pilot schools to use VR for flight training.*	*Perceived enjoyment positively effects perceived behavioural control*	*VR*
McGowin, Xi	*2020*	*Study examining how VR flight simulators could be used for learning efficacy diagnoses, through various forms of knowledge assessment as a predictor of VR simulator performance*	*Trainees with high comprehension of conceptual knowledge demonstrated higher knowledge transfer in the VR simulator.*	*VR*
Ommerli	*2020*	*Study validating and quantifying the effect on situational awareness of immersion, telepresence, and interactivity within a VR flight simulator*	*Telepresence supports situational awareness in VR.*	*VR*
Walden and Pritchard	*2020*	*Presentation on gauging possible VR pilot training ideas, and determining/exploring VR as a viable platform*	*VR allowed for increased communication and enjoyment but was significantly limited by the VR controls.*	*VR, MR*
Schaffernak, Moesl	*2020*	*Survey of opinion of pilot on AR for pilot training, including internal simulation mechanics, with focus on gendered preferences.*	*Women seemingly preferred the achievement of a specified target or receiving points in the game, more so than did men. Men seemingly preferred a story in the game, more so than did women.*	*AR*
Fussell and Truong	*2020*	*Initial validation of a survey instrument for an extended TAM, with exploration of TAM*	*Successfully confirmed the validity and dependability of the new survey. The validation*	*VR*

		factor for intentions toward VR aviation training	*allows for the evaluation of VR flight training, and may be suitable for other xR flight training applications.*	
Oh	*2020*	*Documents two experiments examining perceived difference between VR and conventional training simulators, by both student and instructor pilots.*	*Based on the perceptions of the two group of visibility for checking points for flight, operational tension recreation, and situation awareness in the environment, it appears that increased flight hours require a higher level of fidelity. Observations about the limitations of the implemented VR solution differed between the student and instructor groups as well. Instructors tended to observe software issue, and students tended to observe hardware issues.*	*VR*
Alce, Klang	*2020*	*Development of an AR approach to teaching cockpit flows, assessed by NASA-TLX and SUS.*	*No task-loading score was excessive, though mental demand was higher than most other sub-scores. SUS score was above average, indicating good perceived learnability and ease of use.*	*AR*
Kurkchubasche, Mills-Thysen	*2020*	*Video presentation about the use of a guided online course and an in-person VR simulation.*	*Proposes lower costs, improved immersion, and the potential for such an arrangement to enable unlimited guided practice.*	*VR*
Fussell and Truong	*2021*	*Investigation of the intentions of pilot participants to use VR for training, using a survey.*	*Intention to use VR for dynamic learning is positively influenced by their attitude towards VR, which is influenced by perception of the ease of use and enjoyment, but not by expectation of improved performance.*	*VR*
Weelden, Alimardani	*2021*	*Discusses the state-of-the-art of VR for training, and the application of neurotechnology for objective measurement of training progress.*	*The combination of VR and modern neurotechnology, when used to measure training progress and feedback, may allow for the optimisation of individual learning.*	*VR*

Füchter, Schlichting	*2021*	*Details the development of a concept AR panel trainer.*	*Use of AR as a human interface for training is accepted by the users and can help improve skills.*	*AR*
Alliya, Samuel	*2021*	*Overview and background of a project to examine the use of VR/AR in pilot training, via evaluation of a socio-cognitive nature*	*The use of VR, and using neurocognitive monitoring and analysis, may help identify gaps in trainee preparation.*	*VR, AR*
Auer, Jerken	*2021*	*Study examining the viability of a consumer grade VR headset to replace or supplement a high-end physical flight simulator.*	*Recent consumer-grade VR cannot fully replace PFS for cockpit familiarization training yet. observed Simulator sickness reached problematic levels, despite the fact that the simulated aircraft remained motionless at all times.*	*VR*
Martins, Oliveira	*2021*	*Identification, via literature search and analysis of current solutions, of the requirements for a VR training simulator.*	*Identified requirements, problems and possible solutions for a VR flight simulator and interface for F-16 pilots*	*VR*
Schaffernak, Moesl	*2021*	*Development of an AR application for supporting landing training in a flight simulator setting.*	*Performance of the AR treatment group appears to be highly dependent on correct calibration of the AR device.*	*AR*
Shevchenko	*2021*	*Investigation of the feasibility of completing abnormal operations training in VR*	*Positive feedback from participant regarding the human-centred design approach and the two training modes*	*VR*
Dymora, Kowal	*2021*	*Development of a VR cockpit for training, with survey response to initial experience.*	*Survey of general knowledge of VR. There may be an optimum period of exposure to VR beyond which discomfort is likely. VR may enhance academic performance above that of traditional educational interventions.*	*VR*
Montalbano, Abich	*2021*	*Observation and survey of perceptions of VR training simulator hardware usability metrics*	*Qualification level appears to impact assessment of input hardware validity in a VR simulator.*	*VR*
Lallai, Zedda	*2021*	*Design and implementation of an AR/MR system for real-time identification of*	*Successful creation of an AR/MR architecture that provides systematic and contextualized training*	*AR, MR*

		procedure critical objects by the student pilot, and monitoring of progress for the instructor	*for cockpit procedures, including automatic feedback and detection of trainee progress.*	
Chang-Geun, Lee	*2021*	*Qualitative evaluation of a developed first-person & third-person VR flight training solution.*	*Perceptions of the potential effectiveness was affected by the participants flight hours, with greater indications of limitations by those with greater hours.*	*VR, MR*
Lekea, Stamatelos	*2021*	*Evaluation of a VR "escape room" simulator scenarios for training emergency procedures.*	*A VR escape room, for the training of flight emergencies, would be helpful to trainee pilot, with the possibility to automate the process.*	*VR*
Mirzaagha	*2021*	*Study of VR impact on time-based and evet-based prospective memory, showing that time-based prospective memory was enhanced when tested in VR*	*VR was not generally superior, though time-based prospective memory was improved when tested in a VR environment. It was theorised that improved time-based prospective memory may be due to improved situational awareness in a more immersive situation.*	*VR*
Xie, Sie	*2021*	*Documents the development and initial evaluation of a MR RPAS simulator for drone training, importantly including wind.*	*Evaluation of the developed systems, by questionnaire and interview of drone experts, suggests that such a system would be beneficial for the licencing exam, and that the virtual drone had an adequate degree of physical feedback to make them feel more real.*	*MR*
Abich, Montalbano	*2021*	*Evaluation of, and recommendations for, immersive training devices (ITD), based upon the experiences of flight instructor pilots.*	*Concludes that t the use of xR flight training technology is an area worthy of further investigation*	*VR*
Fussell and Hight	*2021*	*Comparative evaluation of VR and 2D flight training simulation, evaluated with SUS, GUESS, and TLX.*	*Eyestrain was statistically higher in the VR group than the 2D group. No conclusion reached regarding the effectiveness of VR technology for flight training. The participants in the non-VR Group found the manoeuvres easier to perform in the*	*VR*

			simulations than those in the VR Group.	
Hosler	*2021*	*Exploration of the possible benefits of the application of the ADDIE framework to a VR pilot familiarisation program.*	*When designing a course of training, which will use VR technology, it is necessary to consider the inherent features of that technology. The common features of VR are immersion, presence, and interaction. Suggests questions that should be asked during the training design phase.*	*VR*
Labedan, Darodes-De-Tailly	*2021*	*Comparison of VR and real-world flight training effects on student pilots, evaluated with subjective questionnaire and ECG.*	*Real flight conditions induce higher mental demand and psychological stress than in the VR simulator condition.*	*VR*
Livatino, Mohamed	*2022*	*Partially a review of existing research, but with a proposed investigation of a VR system compared to a 2D monitor simulator in terms of presence.*	*Negligible.*	*xR*
Harris, Arthur	*2022*	*Examination of perceived fidelity and eye movements within a VR training simulator.*	*Expert pilots considered the simulation realistic and potentially useful for training, but further development was required for assessment criteria.*	*VR*
Zhang	*2022*	*Thesis investigating the effect of using VR compared to conventional PCATD for training.*	*VR flight simulation could provide a better user experience and generate a higher motivation for usage.*	*VR*
Albeaino, Eiris	*2022*	*Evaluation of comparative task loading (NASA TLX) between VR simulator and real-world training, focussed on drone-mediated building inspection education*	*Except for temporal demand, the TLX ratings showed no statistically significant differences between VR and real-world task loading. Temporal demand was higher, to a statistically significant extent, for the VR group.*	*VR*
Hight, Fussell	*2022*	*Quasi-experimental study evaluating the educational benefits of VR simulator training, with primary evaluation via CBT and simulation objective scores*	*VR did not hinder learning and may have slightly enhanced academic performance – not conclusive.*	*VR*
Guthridge	*2022*	*Evaluation of the efficacy of training on a VR flight*	*Quantitative and qualitative evaluation of*	*VR*

		simulator, as compared to a traditional PCATD, conducted on beginner instrument pilots.	*VR flight simulator effects. VR Improves performance of manoeuvres based on visual cues. Positive response to VR flight simulation.*	
Ross	*2022*	*An analysis of two focus groups on the opinions of flight instructors towards xR for flight training*	*Responses to questioning suggest that there is support of xR in flight training, and for the reduction in cost through this adoption.*	*VR, AR, MR, XR*
Gaurav, Raquel	*2022*	*Evaluation of Galvanic vestibular stimulation (GVS) for the creation of vestibular flight illusion, within a VR flight simulator, with applications to the training of pilots*	*Mismatching galvanic vestibular stimulation can impact the perception of motion, and create flight illusion perception, in a VR flight training simulator.*	*VR*
Morana, Iqbal	*2022*	*Examination of performance differences between VR and traditional display technology - search & find, and RPAS flight task*	*HMD is superior to 2D display (monitor) for difficult training tasks, including better precision and completion time. HMD (VR) also rates well of comfort and presence.*	*VR*
Schaffernak, Moesl	*2022*	*Exploration of possible MR application, based upon professional stakeholder consultation.*	*Identified interactive theory training, cockpit procedure, and outside check training as the most promising applications of AR and MR.*	*AR, MR*
Moesl, Schaffernak	*2022*	*Survey of opinion on difficulties in type rating training, and possible solutions through AR*	*Assessed gender specific preference and difficulties in type-rating training via survey, and the potential for an AR-based solution. Both genders considered an AR-based solution to be potentially beneficial.*	*AR*
Dittel, Matyas	*2022*	*Exploration of a VR flight simulator for transition training between SPL and private pilot licence.*	*VR was not suitable to use as the only means for flight training but is a suitable tool for portions of PPL training. VR training for a PPL should be supplemented by classic training in an actual aircraft.VR was also identified as giving some SPL holders that a false sense of achievement, which could conceivably have dangerous ramifications for safety. There is also a*	*VR*

		suggestion of the development of "compensatory skills" for the landing flair in the VR when combined with improper technique due to previous experience in a different category of aircraft.	
