# Supplementary material for: Applications of extended reality in pilot flight simulator training: a systematic review with meta-analysis
Source: Vis Comput Ind Biomed Art. 2025 Oct 23;8:25. doi: 10.1186/s42492-025-00206-w (PMC12546163; doi:10.1186/s42492-025-00206-w)
Supplement: Supplementary file 2 — Supplementary Material 2. Appendix B. [file 42492_2025_206_MOESM2_ESM.pdf]

Appendix B – Tabulated Thematic Analysis Data

APPLICATIONS AND MOTIVATIONS

	Simulation	Procedure trainer	AR Procedure Guidance	Access Limited Resources	Distance Learning	Embedded AI/ML or Data-driven system	PTN	Constructivism	Collaboration	Gamification	Other Pedagogy	Increased Immersion	Increased Motivation	Increased Enjoyment	Personalised	Deeper Learning
	Application							Pedagogical Motivations				Intrinsic Motivations				
Mowafy 1993	x							x	x			x				x
Vince 1993	x											x				
Amburn 1996											x					
Magee 2000	x							x				x				
Bles 2001	x															
Doerr 2001	x	x	x			x		x	x							
Aka 2002	x				x	x										
Ntuen 2004	x							x				x				
Bauer 2008	x	x						x								
Gu 2009	x			x								x				
Sciarini 2015	x			x	x											
Cardenas 2017	x			x		x		x			x	x				
Brown 2017										x						
Montalvo 2018												x				
Kazenmaier 2018	x			x												
Lawrynczyk 2018	x			x							x	x				
Brown 2018		x	x	x	x	x		x								
Giaimo 2018	x			x	x		x	x								
Gustafsson 2018	x											x				
Biddle 2019	x	x		x		x										
Redei 2019	x										x			x		
Brown 2019				x	x	x		x		x					x	x
Pennington 2019	x			x		x	x	x	x						x	x
Mendonca 2019	x							x				x				
Ommerli 2019	x										x	x				
Bell 2020	x					x	x		x			x				
Salazar 2020		x		x	x			x		x				x		x
Fussell 2020	x											x				x
McGowin 2020	x										x					x
Ommerli 2020	x											x				x
Walden 2020	x							x		x						
Schaffernak 2020	x	x	x	x	x					x						
Fussell 2020	x	x														
Oh 2020	x											x				
Alce 2020	x	x	x					x							x	x
Kurkchubasche 2020	x			x	x			x				x				x
Fussell 2021	x				x											
Weelden 2021						x										
Füchter 2021		x		x	x			x		x				x		x
Alliya 2021	x	x	x			x		x		x			x	x	x	
Auer 2021	x	x						x								
Martins 2021	x			x							x	x				
Schaffernak 2021	x	x						x								
Shevchenko 2021	x	x									x				x	
Dymora 2021																
Montalbano 2021	x															
Lallai 2021	x		x					x	x							
Chang-Geun 2021	x					x			x		x					x
Lekea 2021	x									x		x				
Mmirzaagha 2021	x										x	x				
Xie 2021	x							x								
Abich 2021	x						x									
Fussell 2021	x												x	x		
Hosler 2021	x					x										
Labedan 2021	x							x				x				
Livatino 2022		x									x	x				
Harris 2022	x	x				x		x				x				
Zhang 2022	x	x						x				x	x			
Albeaino 2022	x							x								
Hight 2022	x							x								x
Guthridge 2022	x			x	x	x		x				x				
Ross 2022				x												
Gaurav 2022												x				
Morana 2022	x	x										x				
Schaffernak 2022		x		x												x
Moesl 2022					x									x	x	
Dittel 2022	x							x								
**Total**	**53**	**18**	**6**	**18**	**12**	**14**	**4**	**28**	**6**	**8**	**11**	**26**	**3**	**6**	**6**	**13**
	125							53				54				

xR Spectrum and Technology

	VR	AR	MR	XR	HTC Vive Pro	Oculus/Meta	Smartphone/Tablet	Microsoft Hololens	Custom or unusual Hardware	No hardware specified
	xR Spectrum				xR Technology					
Mowafy 1993	x									x
Vince 1993	x									x
Amburn 1996	x								x	
Magee 2000	x								x	
Bles 2001	x									x
Doerr 2001	x		x							x
Aka 2002	x									x
Ntuen 2004	x									x
Bauer 2008	x								x	
Gu 2009			x						x	
Sciarini 2015		x							x	
Cardenas 2017			x	x					x	
Brown 2017	x	x	x					x	x	
Montalvo 2018		x						x		
Kazenmaier 2018	x				x					
Lawrynczyk 2018	x					x				
Brown 2018		x	x					x		
Giaimo 2018	x									x
Gustafsson 2018	x					x				
Biddle 2019	x								x	
Redei 2019	x					x				
Brown 2019		x						x		
Pennington 2019	x									x
Mendonca 2019	x									x
Ommerli 2019	x					x				
Bell 2020	x				x					
Salazar 2020		x					x			
Fussell 2020	x									x
McGowin 2020	x				x					
Ommerli 2020	x					x				
Walden 2020	x		x			x				
Schaffernak 2020		x								x
Fussell 2020	x									x
Oh 2020	x					x			x	
Alce 2020		x						x		
Kurkchubasche 2020	x				x					
Fussell 2021	x									
Weelden 2021	x									x
Füchter 2021		x					x			
Alliya 2021	x	x								x
Auer 2021	x								x	
Martins 2021	x									x
Schaffernak 2021		x						x		
Shevchenko 2021	x				x					
Dymora 2021	x				x					
Montalbano 2021	x									x
Lallai 2021		x	x					x		
Chang-Geun 2021	x		x						x	
Lekea 2021	x									x
Mmirzaagha 2021	x				x					
Xie 2021			x							x
Abich 2021	x									x
Fussell 2021	x				x					
Hosler 2021	x									x
Labedan 2021	x				x					
Livatino 2022					x					
Harris 2022	x								x	
Zhang 2022	x				x					
Albeaino 2022	x					x				
Hight 2022	x				x					
Guthridge 2022	x				x					
Ross 2022	x	x	x	x					x	
Gaurav 2022	x					x				
Morana 2022	x					x				
Schaffernak 2022		x	x							
Moesl 2022		x								x
Dittel 2022	x								x	
**Total**	**51**	**15**	**11**	**2**	**13**	**10**	**2**	**7**	**14**	**21**
	79				67					

Domain & Level of Training

	Recreational	RPAS	GA	Airline	Military	Familiarisation	Ab initio	General Handling	Procedure	Type Training	Phase/Action/Maneuver	Systems	Remotely Piloted Aircraft System	NOT SPECIFIED/DETERMINABLE
	Domain					Level - General			Level - Specific					
Mowafy 1993					x						x			
Vince 1993														
Amburn 1996					x									
Magee 2000					x						x			
Bles 2001														x
Doerr 2001				x					x			x		
Aka 2002														x
Ntuen 2004							x	x						
Bauer 2008				x		x			x	x	x			
Gu 2009			x											
Sciarini 2015					x	x								
Cardenas 2017								x						x
Brown 2017											x			
Montalvo 2018														x
Kazenmaier 2018														
Lawrynczyk 2018	x		x				x	x						
Brown 2018			x	x		x			x		x			
Giaimo 2018					x									
Gustafsson 2018														x
Biddle 2019				x							x			
Redei 2019					x						x			
Brown 2019				x										
Pennington 2019					x		x							
Mendonca 2019											x			x
Ommerli 2019			x	x										
Bell 2020					x									
Salazar 2020			x			x						x		
Fussell 2020														x
McGowin 2020														x
Ommerli 2020														x
Walden 2020			x		x			x	x					
Schaffernak 2020			x	x		x			x		x			
Fussell 2020														
Oh 2020			x											
Alce 2020				x		x			x	x	x			
Kurkchubasche 2020			x			x	x				x			
Fussell 2021			x	x			x	x			x			
Weelden 2021														x
Füchter 2021			x			x						x		
Alliya 2021			x	x		x	x	x	x					
Auer 2021				x		x		x	x		x			
Martins 2021					x									
Schaffernak 2021			x								x			
Shevchenko 2021									x					
Dymora 2021														
Montalbano 2021					x									x
Lallai 2021				x							x			
Chang-Geun 2021														x
Lekea 2021					x						x			
Mmirzaagha 2021									x					
Xie 2021						x							x	
Abich 2021					x									
Fussell 2021			x				x							
Hosler 2021					x	x								
Labedan 2021			x				x	x						
Livatino 2022						x						x		
Harris 2022				x				x	x	x				
Zhang 2022			x					x			x			
Albeaino 2022		x									x		x	
Hight 2022			x	x			x							
Guthridge 2022			x				x	x			x			
Ross 2022														x
Gaurav 2022														x
Morana 2022		x									x		x	x
Schaffernak 2022						x					x	x		
Moesl 2022				x						x				
Dittel 2022	x		x			x	x	x						
**Total**	**2**	**2**	**19**	**15**	**14**	**15**	**11**	**14**	**11**	**4**	**21**	**5**	**3**	**15**
	52					40			59					

Skills

	NTS1.1 – Maintain effective lookout	NTS1.2 – Maintain situational awareness	NTS1.3 – Assess situations and make decisions	NTS1.4 – Set priorities and manage tasks	NTS1.5 – Maintain effective communications and interpersonal relationships	NTS2.1 – Recognise and manage threats	NTS2.2 – Recognise and manage errors	NTS2.3 – Recognise and manage undesired aircraft state	A1.1 – Start and stop engine	A2.1 – Carry out pre-take-off procedures	A2.2 – Take off aeroplane	A4.1 – Land aeroplane	A4.2 – Land aeroplane in a cross-wind	A3.6 – Perform circuits and approaches	A3 - Control aeroplane in normal flight	A3.1 – Climb aeroplane	A3.4 – Turn aeroplane	A3.2 – Maintain straight and level flight	A6.3 – Perform forced landing (simulated)	A6.5 – Manage other abnormal situations (simulated)	TR-SEA.10 – Manage partial and complete engine failure situations in flight	H2.1 – Lift-off and hover helicopter, perform hover checks and abort take-off	H2.6 – Land from the hover	H6.4 – Land, take off and manoeuvre in a confined area	Perform turns around nose and tail	H2.5 – Perform sideways and backwards flight	C2.1 – Pre-flight actions and procedures	C2.2 – Perform pre-flight inspection	C1.2 – Operational communication using an aeronautical radio	MCO.1 – Operate effectively as a crew member	MCO.2 – Demonstrate effective leadership and authority	FAE-6.1 – Fly echelon formation	SIR.4 – Monitor learning in a flight simulation training device environment	RM3 - Advanced manoeuvres
	NTS								Rating Standard																		Common Standard					Other		
Mowafy 1993		x	x			x																											x	
Vince 1993																																		
Amburn 1996		x																																
Magee 2000																						x	x	x										
Bles 2001																																		
Doerr 2001																																		
Aka 2002																																		
Ntuen 2004											x	x		x	x	x	x																	
Bauer 2008									x																		x		x					
Gu 2009																																		
Sciarini 2015																																		
Cardenas 2017																																		
Brown 2017																																		
Montalvo 2018																																	x	
Kazenmaier 2018																						x			x	x								
Lawrynczyk 2018		x									x	x		x		x	x	x																
Brown 2018																											x	x						
Giaimo 2018																																		
Gustafsson 2018																																		
Biddle 2019																														x	x			
Redei 2019															x																	x		
Brown 2019																																		
Pennington 2019																																		
Mendonca 2019	x	x		x		x																												
Ommerli 2019		x			x																								x					
Bell 2020																																		
Salazar 2020		x							x	x																								
Fussell 2020																																		
McGowin 2020																																		
Ommerli 2020		x																																
Walden 2020	x	x			x		x				x																		x					
Schaffernak 2020																																		
Fussell 2020																																		
Oh 2020		x	x			x	x	x				x	x						x	x														
Alce 2020										x																x								
Kurkchubasche 2020																																		
Fussell 2021																																		
Weelden 2021																																		
Füchter 2021		x							x	x																								
Alliya 2021																																		
Auer 2021																												x						
Martins 2021																																		
Schaffernak 2021												x																						
Shevchenko 2021																				x														
Dymora 2021									x																									
Montalbano 2021																																		
Lallai 2021											x																							
Chang-Geun 2021																																		
Lekea 2021																																		
Mmirzaagha 2021																																		
Xie 2021	x																																	x
Abich 2021																																		
Fussell 2021																																		
Hosler 2021																																		
Labedan 2021			x								x	x	x	x		x	x	x																
Livatino 2022		x																																
Harris 2022									x	x		x																						
Zhang 2022		x													x	x	x	x																
Albeaino 2022																																		x
Hight 2022																																		
Guthridge 2022	x	x			x									x	x		x	x																
Ross 2022																																		
Gaurav 2022																																		
Morana 2022	x	x	x					x																										x
Schaffernak 2022		x																										x						
Moesl 2022																																		
Dittel 2022		x	x						x	x	x	x		x	x	x	x	x																
**Total**	5	16	5	1	3	3	2	2	6	5	6	7	2	5	5	5	6	5	1	2	0	2	1	1	1	2	2	3	3	1	1	1	2	3
	37								62																		10					6		

Limitations

	Overhead		Input Problems				Rating Standard							Usefulness		
	COST	TIME	INPUT HARDWARE USABILITY	RECOGNITION INACCURACY	LACK OF FEEDBACK	PHYSICAL OR DESIGN LIMITATION	HMD LIMITATION	SOFTWARE LIMITATION	SOFTWARE USABILITY	MOTION SICKNESS OR OPERATOR HARM	FIELD OF VIEW	COMPENSATORY	PHYSICAL OR DESIGN LIMITATION	LACK OF REALISM	NO REPORTED ISSUES	INEFFECTIVE
Mowafy 1993																x
Vince 1993	x	x		x			x			x						
Amburn 1996											x			x		x
Magee 2000																
Bles 2001										x						
Doerr 2001																
Aka 2002							x									
Ntuen 2004																x
Bauer 2008			x										x			
Gu 2009																
Sciarini 2015													x			
Cardenas 2017																
Brown 2017			x	x									x			
Montalvo 2018							x			x		x				
Kazenmaier 2018			x					x					x	x		
Lawrynczyk 2018										x						x
Brown 2018																
Giaimo 2018																
Gustafsson 2018			x		x	x	x			x	x	x	x			
Biddle 2019															x	
Redei 2019										x					x	
Brown 2019																x
Pennington 2019																
Mendonca 2019																
Ommerli 2019																
Bell 2020																
Salazar 2020															x	
Fussell 2020										x						
McGowin 2020																
Ommerli 2020																
Walden 2020			x	x		x		x	x				x			
Schaffernak 2020																
Fussell 2020																
Oh 2020			x	x			x	x			x	x	x	x		
Alce 2020			x		x		x	x			x		x			
Kurkchubasche 2020																
Fussell 2021																
Weelden 2021															x	
Füchter 2021															x	
Alliya 2021																
Auer 2021			x	x		x				x		x				x
Martins 2021																
Schaffernak 2021			x	x				x	x	x						
Shevchenko 2021		x														
Dymora 2021			x							x						
Montalbano 2021			x													
Lallai 2021						x	x			x	x		x			
Chang-Geun 2021							x					x		x		
Lekea 2021																
Mmirzaagha 2021																
Xie 2021															x	
Abich 2021						x							x			
Fussell 2021										x						
Hosler 2021																
Labedan 2021												x		x		
Livatino 2022																
Harris 2022														x		x
Zhang 2022																
Albeaino 2022			x				x	x								
Hight 2022			x				x	x	x							x
Guthridge 2022			x				x	x					x			
Ross 2022	x						x			x			x	x		
Gaurav 2022										x						x
Morana 2022															x	
Schaffernak 2022	x									x						x
Moesl 2022																
Dittel 2022										x		x				x
**Total**	**3**	**2**	**14**	**6**	**2**	**5**	**12**	**8**	**3**	**16**	**5**	**7**	**12**	**7**	**7**	**11**
	5		27				63							25		
