# Supplementary material for: Applications of extended reality in pilot flight simulator training: a systematic review with meta-analysis
Source: Vis Comput Ind Biomed Art. 2025 Oct 23;8:25. doi: 10.1186/s42492-025-00206-w (PMC12546163; doi:10.1186/s42492-025-00206-w)
Supplement: Supplementary file 3 — Supplementary Material 3. Appendix C. [file 42492_2025_206_MOESM3_ESM.pdf]

Appendix C – Quality of trials included in meta-analysis (CONSORT)

	Auer et al., 2021	Hight et al., 2022	Zhang, 2022	Redei, 2019	Guthridge, 2022
Was the study population adequately described?	yes	yes	yes	yes	yes
Was the minimum important difference described?	not stated	not stated	not stated	not stated	not stated
Was the target sample size adequately determined?	not stated	not stated	not stated	not stated	not stated
Was the unit of randomization described?	not stated	not stated	individual	not stated	not stated
Was intention-to-treat analysis used?	not stated	not stated	not stated	not stated	not stated
Were the participants allocated using random number tables, coin flip, computer generation?	no	no	no	no	no
Was the randomization process concealed from the investigators?	not stated	not stated	not stated	not stated	not stated
Were follow-up measures administered blind?	no	unclear	unclear	no	no
Was estimated effect on primary and secondary outcome measures stated?	yes	yes	yes	yes	yes
Was precision of effect size estimated (confidence intervals)?	no	no	no	no	no
Were summary data presented in sufficient detail to permit alternative analyses or replication?	yes	yes	yes	yes	yes
Was the discussion of the study findings consistent with the data?	yes	yes	yes	yes	yes
